# CDK1 and CEP97 cooperatively control centriole length to orchestrate ciliogenesis and developmental patterning

**DOI:** 10.1101/gad.353426.125

**Published:** 2026-07-01

**Authors:** Yue Liu, Zhengmao Wang, Tanvi Sinha, Kathy H. Li, Robert J. Chalkley, Vicente Herranz-Pérez, Chang Xie, Bradley K. Yoder, Alma L. Burlingame, Brian L. Black, Jeremy F. Reiter

**Affiliations:** 1 Department of Biochemistry and Biophysics, Cardiovascular Research Institute, University of California, San Francisco, San Francisco, California 94158, USA;; 2 Department of Pharmaceutical Chemistry, University of California, San Francisco, San Francisco, California 94158, USA;; 3 Laboratory of Comparative Neurobiology, Institute Cavanilles, University of Valencia, Network Center for Biomedical Research in Neurodegenerative Diseases (CIBERNED)-Carlos III Health Institute (ISCIII), Paterna 46980, Spain;; 4 Department of Cell Biology, Functional Biology, and Physical Anthropology, University of Valencia, CIBERNED-ISCIII, Burjassot 46100, Spain;; 5 Department of Cell, Developmental, and Integrative Biology, University of Alabama at Birmingham, Birmingham, Alabama 35294, USA;; 6 Chan Zuckerberg Biohub, San Francisco, California 94158, USA

**Keywords:** CEP97, Centrobin, Hedgehog signaling, cell cycle, centriole, cyclin-dependent kinase 1, developmental patterning, phosphoproteomics, phosphorylation, primary cilia

## Abstract

Architecture is critical for organelle function. The lengths of centrioles, key components of centrosomes, are tightly regulated. We adapted centrosome purification approaches to measure centrosomal protein phosphorylation. Centrosome-specific phosphoproteomics with and without cyclin-dependent kinase 1 (CDK1) activity revealed that CDK1 phosphorylates many centriolar proteins involved in centriole length control, including Centrobin. CDK1 regulated centriole length synergistically with CEP97-CCP110, a local centriolar complex. CEP97 restricted Centrobin localization to centrioles, whereas CDK1-dependent phosphorylation suppressed Centrobin's ability to promote centriole elongation. Thus, CDK1 and CEP97-CCP110 both restrict centriole elongation by inhibiting the function of Centrobin, a centriole elongation factor, but via different mechanisms. Overelongated centrioles failed to support ciliogenesis in human cells and in mouse embryos. Removing CEP97 during mouse development caused centriole overelongation, impaired ciliogenesis and attenuated Hedgehog (HH) signaling, disrupting mouse heart development. We conclude that CEP97-CCP110 and CDK1 cooperatively restrict Centrobin function to control centriole length, critical for mammalian development.

## Abstract (precis)

In this study, Liu et al. performed centrosome-specific phosphoproteomics and reveal that CDK1 and CEP97 synergistically restrict the function and localization of Centrobin (a centriole elongation factor) and thus control centriole length. This mechanism is critical for ciliogenesis, mitotic progression, and hedgehog signaling, which together regulate mammalian heart and brain development.

Cells regulate the size of their organelles (Marshall 2002). The centrosome is a membrane-less organelle that organizes cytoplasmic microtubules and supports the primary cilium (Mill et al. 2023; Laporte et al. 2024). At the heart of the centrosome is a pair of centrioles (Fernandes-Mariano et al. 2025). Centrioles are highly ordered cylindrical structures comprised of nine triplet microtubules that polymerize at their plus ends (Wong and Stearns 2003; Shukla et al. 2015; Aydogan et al. 2018, 2020; Marteil et al. 2018; Nigg and Holland 2018; Sullenberger et al. 2020; Blanco-Ameijeiras et al. 2022). While different cell types can have centrioles of different lengths, there is little variance within a cell type, suggesting tight control of length (Fernandes-Mariano et al. 2025). Centriolar proteins such as CEP97, CCP110, OFD1, MNR, and Centrobin all help control centriole length (Spektor et al. 2007; Kohlmaier et al. 2009; Korzeniewski et al. 2010; Singla et al. 2010; Gudi et al. 2011, 2015; Kumar et al. 2021; Iyer et al. 2025). In particular, Centrobin dynamically localizes to centrioles to promote centriole elongation, coordinate centriole maturation and support mitotic spindle formation (Zou et al. 2005; Jeffery et al. 2010; Gudi et al. 2011, 2015; Park and Rhee 2013; Le Roux-Bourdieu et al. 2022; Lee et al. 2025).

Phosphorylation regulates centrosome and centriole biogenesis, which occurs once and only once per cell cycle at the beginning of S phase (Fu et al. 2015; Nigg and Holland 2018; Fernandes-Mariano et al. 2025). Centrosome and centriole proteins are phosphorylated by several kinases. For example, PLK4 phosphorylates itself, STIL and CPAP (also known as CENPJ and SAS-4) to initiate centriole biogenesis (Novak et al. 2016; Dzhindzhev et al. 2017; McLamarrah et al. 2018; Ohta et al. 2018; Moyer and Holland 2019; Scott et al. 2023), and PLK1 and NEK2 phosphorylate Centrobin to regulate its centriolar localization and control of microtubule stability (Park and Rhee 2013; Le Roux-Bourdieu et al. 2022). Additional kinases, including cyclin-dependent kinase 1 (CDK1), coordinate centriole maturation and disengagement (Chen et al. 2002; Hochegger et al. 2007; Wang et al. 2014; Novak et al. 2016; Zitouni et al. 2016; Huang et al. 2022; Steinacker et al. 2022; Roberts et al. 2024). While CDK1 advances the cell cycle into mitosis (Santamaría et al. 2007; Hochegger et al. 2008; Pellarin et al. 2025), it also operates independently of the cell cycle to regulate transcription, translation and mitochondrial function (Taguchi et al. 2007; Haneke et al. 2020; Enserink and Chymkowitch 2022; Massacci et al. 2023).

The centriole is an extremely large (>1600 MDa), highly stable macromolecular complex comprised of >100 proteins, almost all of which occur in many copies (Loncarek and Bettencourt-Dias 2018; LeGuennec et al. 2021). The size and complexity of the centriole have made understanding how the centriole is built and functions difficult. Centrosome affinity capture (CAPture)-mass spectrometry (MS), developed by Carden et al. (2023), biochemically purifies the centrosome, allowing for centrosome proteome identification with high specificity.

Overlapping functions of genes and pathways provide a mechanism by which biology ensures robustness and fidelity. For example, multiple DNA repair pathways maintain genome integrity (Lanz et al. 2019). In this study, we develop centrosome-specific phosphoproteomics and identify overlapping functions of CDK1 and CEP97-CCP110 in restraining Centrobin function and centriole elongation, key for mammalian heart development.

## Results

### Characterization of the centrosomal phosphoproteome

**Figure 1.**
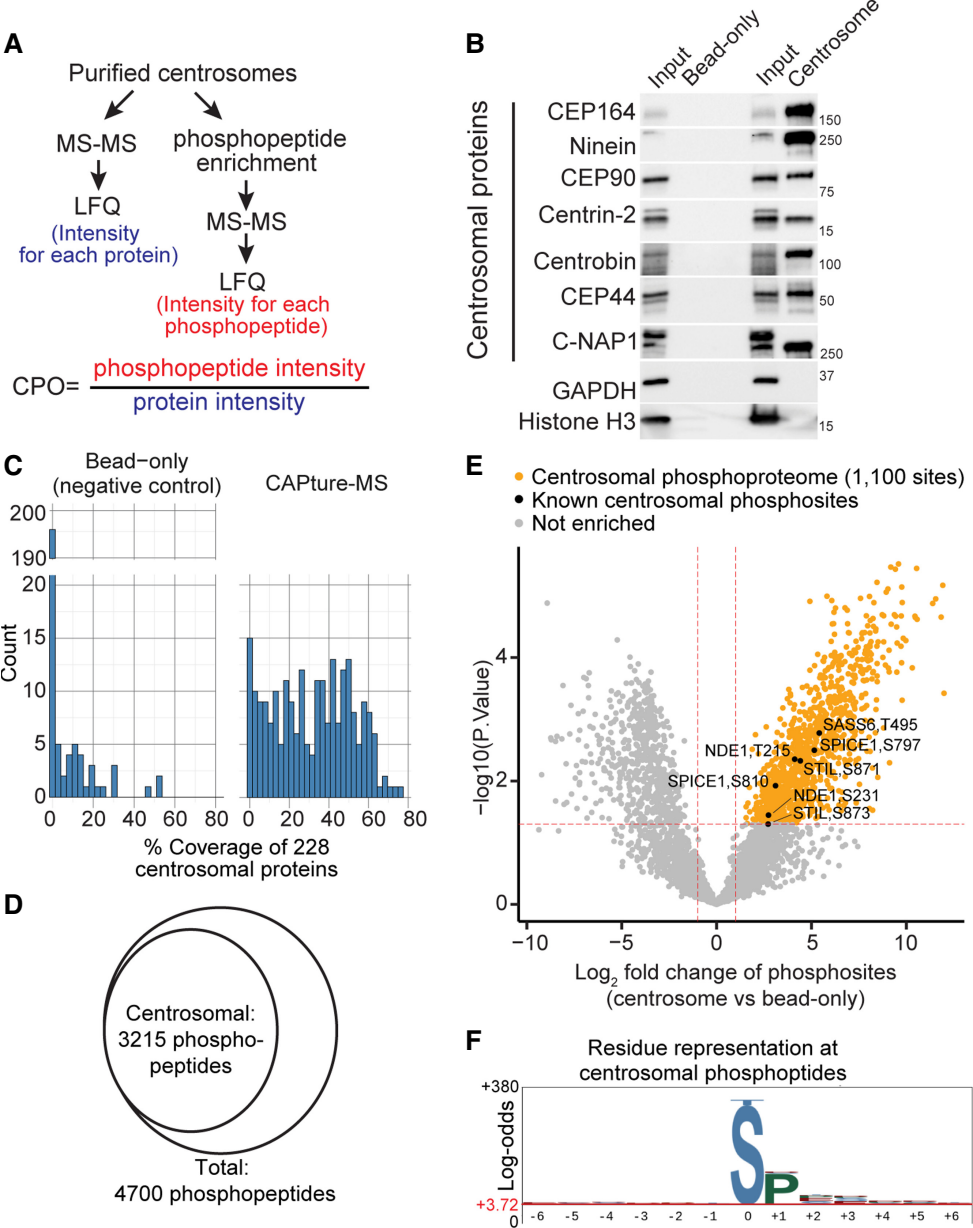
Profile of the centrosomal phosphoproteome. (*A*) A schematic of the workflow. Centrosomes were purified from Expi293F cells with CAPture. Five percent of isolated centrosomes were directly processed for mass spectrometry (MS) analysis. The rest was enriched for phosphopeptide using Fe-nitrilotriacetic acid-immobilized metal ion affinity chromatography before processing for MS analysis. Centrosomal phosphopeptide occupancy (CPO) is the ratio of phosphopeptide intensity to protein intensity. *n* = 3 for each condition. (*B*) CAPture purified centrosomes from Expi293F cells. Purified centrosomes and bead-only (negative control) were immunoblotted for indicated proteins. (*C*) Histograms of the coverage of centrosomal proteins identified using CAPture-MS and bead-only. (*D*) Venn diagram showing that of the CAPture-phosphoMS-identified unique phosphopeptides, 3215 (68%) phosphopeptides mapped to known centrosomal proteins. (*E*) Volcano plot of differentially phosphorylated residues comparing CAPture-phosphoMS enriched phosphopeptides to those detected using bead-only control. Vertical dotted red lines indicate plus or minus twofold change, and horizontal dotted red lines indicate *P*-values of 0.05. Orange dots indicate 1100 CAPture-phosphoMS-identified phosphosites. Select phosphorylated proteins with known functions in centrosome biology are labeled. (*F*) One-thousand-one-hundred centrosomal phosphosites, identified as in *E*, were used to generate motif visualizations with pLogo.

To investigate the phosphorylation of centrosomal proteins, we combined affinity purification of centrosomes with mass spectrometric phosphoproteomics (Carden et al. 2023). This approach, which we call CAPture-phosphoMS, built upon a previously developed technique for centrosome affinity capture (CAPture) by adding phosphopeptide enrichment with immobilized metal ion affinity chromatography and identification with tandem MS analysis (phosphoMS) (Fig. 1A; Carden et al. 2023). Consistent with previous work, CAPture-MS detected 228 previously identified centrosomal proteins, with ∼31% (95% CI: 29.2%–32.2%) protein coverage, indicating successful purification of the centrosome (Fig. 1B,C; Carden et al. 2023). After phosphopeptide enrichment, CAPture-phosphoMS detected 4700 unique phosphopeptides (Supplemental Table S1).

Of these, 3215 (68%) unique phosphopeptides mapped to 160 previously identified centrosomal proteins, indicating that CAPture-phosphoMS enriched for centrosomal phosphopeptides (Fig. 1D). After accounting for peptides with multiple phosphorylations, CAPture-phosphoMS identified 1100 phosphosites distributed across 954 serines, 139 threonines, and seven tyrosines (Fig. 1E; Supplemental Table S2). These findings indicate that centrosomal proteins are predominantly phosphorylated on serine and threonine, with low levels of tyrosine phosphorylation. A number of the detected centrosomal protein phosphorylations were previously implicated in centrosome duplication, centrosome maturation and spindle assembly (Supplemental Table S3; Hirohashi et al. 2006; Lee and Rhee 2011; Bradshaw et al. 2013; Kratz et al. 2015; Wynne and Vallee 2018; Deretic et al. 2019). Motif analysis of the centrosomal phosphosites revealed enrichment of the motif S/TP, suggesting that centrosomal proteins may be regulated by proline-directed kinases, such as CDK1 (Fig. 1F; Peter et al. 1990; O'Shea et al. 2013; Petrone et al. 2016).

### CDK1-dependent phosphorylation of centrosomal proteins involved in centriole length control

CDK1 functions at the centrosome to regulate centriole duplication, centrosome separation and centriole disengagement (Hochegger et al. 2007; Smith et al. 2011; Novak et al. 2016; Zitouni et al. 2016; Huang et al. 2022). To uncover how CDK1 participates in centrosomal protein phosphorylation, we used CAPture-phosphoMS in combination with CDK1 inhibitor RO-3306 or DMSO vehicle control (Supplemental Fig. S1A–C). We chose to use RO-3306, as it has ∼10-fold selectivity for CDK1 over CDK2 and >50-fold selectivity over CDK4 (Vassilev et al. 2006).

**Figure 2.**
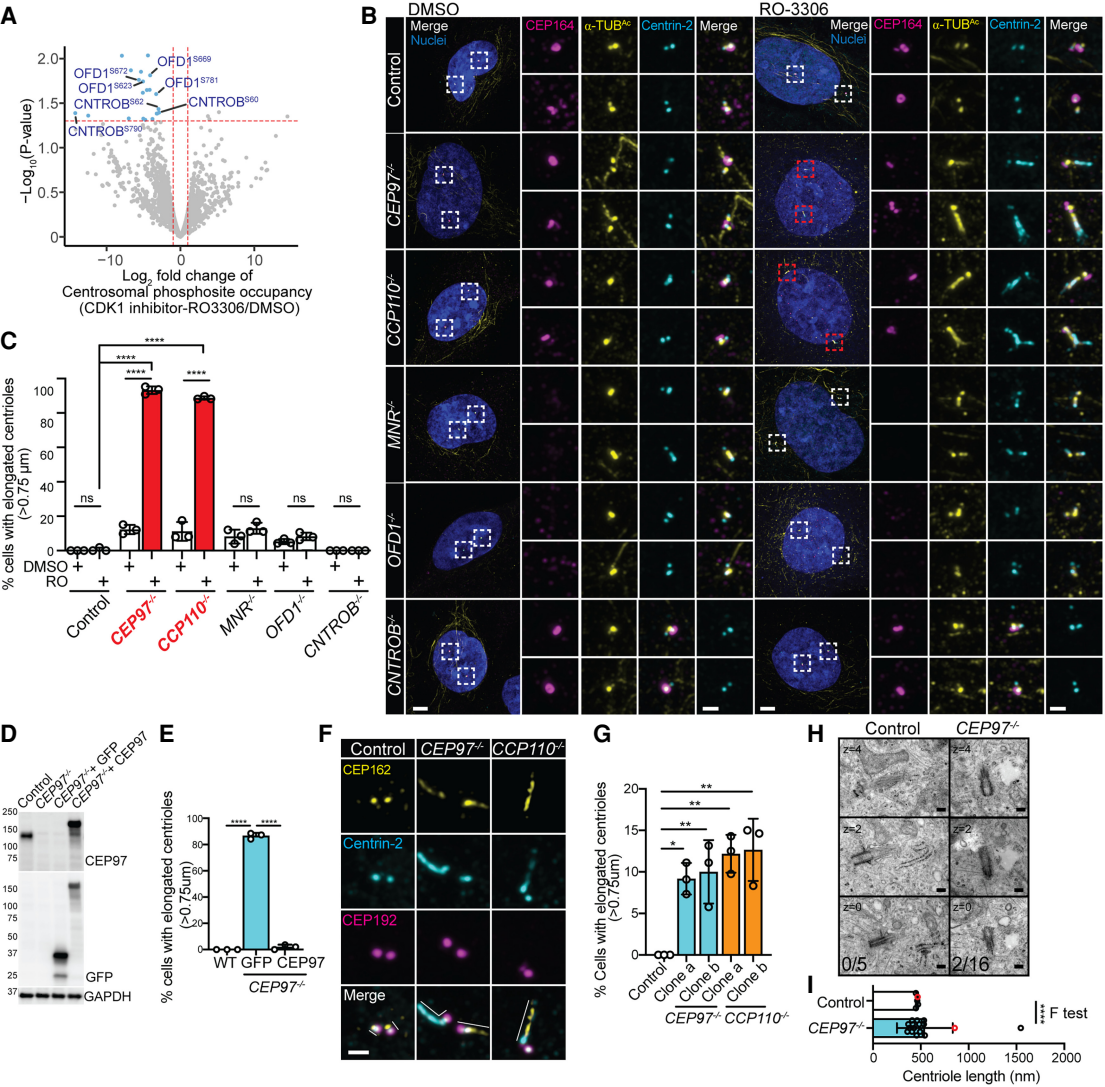
CDK1 synergistically regulates centriole length with CEP97-CCP110. (*A*) Volcano plot of differentially phosphorylated residues comparing the centrosomal phospho-proteome of RO-3306- and DMSO-treated Expi293F cells. Vertical dotted red lines indicate plus or minus twofold change, and horizontal dotted red lines indicate *P*-values of 0.05. CDK1-regulated proteins are colored in cyan. Proteins with known functions in centriole length regulation are labeled. (*B*) Immunofluorescence images of wild-type and indicated mutant RPE1 cells treated with DMSO, or RO-3306 (CDK1 inhibitor) for 24 h and immunostained for CEP164 (mother centriole, magenta), α-Tub^AC^ (centriole; yellow), and Centrin-2 (centrioles; cyan). *Insets* in the *right* panel show magnification of centrioles in boxed regions. Scale bars: 5 µm; *insets*, 1 µm. (*C*) Quantification of the percentage of cells with elongated centrioles in wild-type and indicated mutant RPE1 cells, drug-treated and stained as in *B*. *n* = 3 biological replicates, with 50–100 cells per replicate. (*D*) Immunoblots of wild-type and *CEP97*^*–/–*^ RPE1 cells stably expressing GFP or CEP97-GFP and immunoblotted for indicated proteins. (*E*) Quantification of the percentage of elongated centrioles in wild-type, *CEP97*^*–/–*^ and *CEP97*^*–/–*^ stably expressing CEP97-GFP RPE1 cells treated with RO-3306 for 24 h, as stained in Supplemental Figure S4B. *n* = 3 biological replicates, with 50–100 cells per replicate. (*F*) Immunofluorescence images of wild-type, *CEP97*^*–/–*^ and *CCP110*^*–/–*^ RPE1 cells, stained for CEP162 (yellow), Centrin-2 (cyan), and CEP192 (magenta). Scale bar, 1 µm. (*G*) Quantification of the percentage of cells with elongated centrioles in wild-type, *CEP97*^*–/–*^ and *CCP110*^*–/–*^ RPE1 cells as in *F*. *n* = 3 biological replicates, with 50–100 cells per replicate. (*H*) Serial section TEM of wild-type and *CEP97*^*–/–*^ RPE1 cell centrioles. Fraction of cells with elongated centrioles are shown at the *bottom*. *n* = 5 for wild-type and *n* = 16 for *CEP97*^*–/–*^ centrioles. Scale bars, 200 nm. (*I*) Length of wild-type and *CEP97*^*–/–*^ RPE1 cell centrioles, measured from TEMs as in *H*. Centrioles color coded in red are shown in *H*. Statistical significance was assessed by one-way ANOVA followed by Tukey's multiple comparison tests (*E*,*G*), by two-way ANOVA followed by Šídák's multiple comparison test (*C*), or by *F*-test of equal variance (*I*). A *P*-value of<0.05 was considered statistically significant. (*) *P* < 0.05, (**) *P* < 0.01, (****) *P* < 0.0001. Data are represented as means ± SD.

We calculated the enrichment of each CAPture-phosphoMS-identified phosphorylated residue relative to the summed intensity of its corresponding protein with and without CDK1 inhibition (Fig. 2A; Rodríguez-Ulloa et al. 2024). CDK1 inhibition decreased phosphorylation at 21 peptides that correspond to 14 centrosomal proteins (Fig. 2A; Supplemental Table S4; Bauer et al. 2016; Carden et al. 2023). Several of these 14 CDK1-regulated centrosomal proteins regulate centriole duplication and centrosome separation, processes in which CDK1 participates (Supplemental Table S5; Osmani et al. 2006; Graser et al. 2007; Singla et al. 2010; Gudi et al. 2011; Inanç et al. 2013; Sonnen et al. 2013; Fang et al. 2014; Kim et al. 2015, 2019; Airik et al. 2016; Fu et al. 2016; Park et al. 2018; Kumar et al. 2021; Sullenberger et al. 2023; Theile et al. 2023; Tillery et al. 2024; Lee et al. 2025). Among these, CDK1 promoted the phosphorylation of multiple sites on OFD1 and Centrobin, two regulators of centriole length (Zou et al. 2005; Singla et al. 2010; Gudi et al. 2011, 2015; Lee et al. 2025). Thus, CAPture-phosphoMS suggests that CDK1 phosphorylates diverse centrosomal proteins including regulators of centriole length. We hypothesized that CDK1 may contribute to regulation of centriole length.

### CDK1 functions with CEP97 and CCP110 to regulate centriole length

To test the hypothesis that CDK1 regulates centriole length, we inhibited CDK1 with RO-3306 in RPE1 cells. CDK1 inhibition arrested cell cycle progression, as expected, and did not alter centriole length (Supplemental Fig. S2A,B). We hypothesized that CDK1 may function together with other regulators of centriole length. To test this hypothesis, we inhibited CDK1 in RPE1 cells lacking key centriole length regulators, including OFD1, Centrobin (CNTROB), MNR (also known as KIAA0753 and OFIP), CEP97 and CCP110 (Supplemental Fig. S3A–F). Strikingly, CDK1 inhibition increased centriole length in *CEP97*^*–/–*^ and *CCP110*^*–/–*^ cells, but not in *OFD1*^*–/–*^, *MNR*^*–/–*^, or *CNTROB*^*–/–*^ RPE1 cells (Fig. 2B,C). These results indicate that CDK1 synergistically regulates centriole length together with CEP97 and CCP110.

To confirm that the centriole overelongation phenotype observed in *CEP97*^*–/–*^ cells was due to loss of CEP97, we re-expressed GFP-tagged CEP97 (Fig. 2D). When expressed under the control of a strong promoter, CEP97 does not localize to centrioles (Spektor et al. 2007). We found that when expressed under the control of a weak promoter, CEP97-GFP did localize to centrioles (Supplemental Fig. S4A,B; Ye et al. 2018). CEP97-GFP restored centriole overelongation in CDK1-inhibited *CEP97*^*–/–*^ cells (Fig. 2E; Supplemental Fig. S4B). Thus, CEP97 acts with CDK1 to regulate centriole length.

At high concentrations, RO-3306 can inhibit CDK2, whose function overlaps with CDK1 in centrosome duplication (Hochegger et al. 2007). To determine whether CDK2 also restricts centriole length, we treated control and *CEP97*^*–/–*^ RPE1 cells with K03861, a selective CDK2 inhibitor (Alexander et al. 2015; Coxon et al. 2017; Gemble et al. 2022; Kozyrska et al. 2022). In contrast to CDK1 inhibition, CDK2 inhibition did not alter centriole length in control or *CEP97*^*–/–*^ cells (Supplemental Fig. S5A,B), indicating that CDK2 does not regulate centriole length by itself.

CEP97 and CCP110 physically interact and function together (Spektor et al. 2007). We assessed whether CEP97 and CCP110 localization to the centriole depended on each other. CCP110 failed to localize to centrioles in *CEP97*^*–/–*^ cells (Supplemental Fig. S6A), consistent with previous findings (Spektor et al. 2007). Conversely, CEP97 failed to localize to centrioles in *CCP110*^*–/–*^ cells (Supplemental Fig. S6B). Thus, CEP97 and CCP110 depend on each other for localization to centrioles (Spektor et al. 2007). Furthermore, CCP110 coimmunoprecipitated with CEP97, consistent with previous findings (Supplemental Fig. S6C; Spektor et al. 2007). Immunoprecipitating CEP97 largely depleted CCP110 from the cell lysate (Supplemental Fig. S6C), suggesting that most CCP110 is bound to CEP97.

Without CDK1 inhibitor, removing CEP97 or CCP110 resulted in overelongated centrioles in a minority (∼10%) of cells (Fig. 2F,G). Serial transmission electron microscopy (TEM) confirmed that *CEP97*^*–/–*^ RPE1 cells contained abnormally long centrioles (Fig. 2H,I). Inhibiting CDK1 in *CEP97*^*–/–*^ or *CCP110*^*–/–*^ cells increased the proportion of cells with overelongated centrioles eightfold (Fig. 2B,C). These results indicate that both CEP97 and CCP110 act with CDK1 to restrict centriole length.

### CDK1 can restrict centriole length in S phase

**Figure 3.**
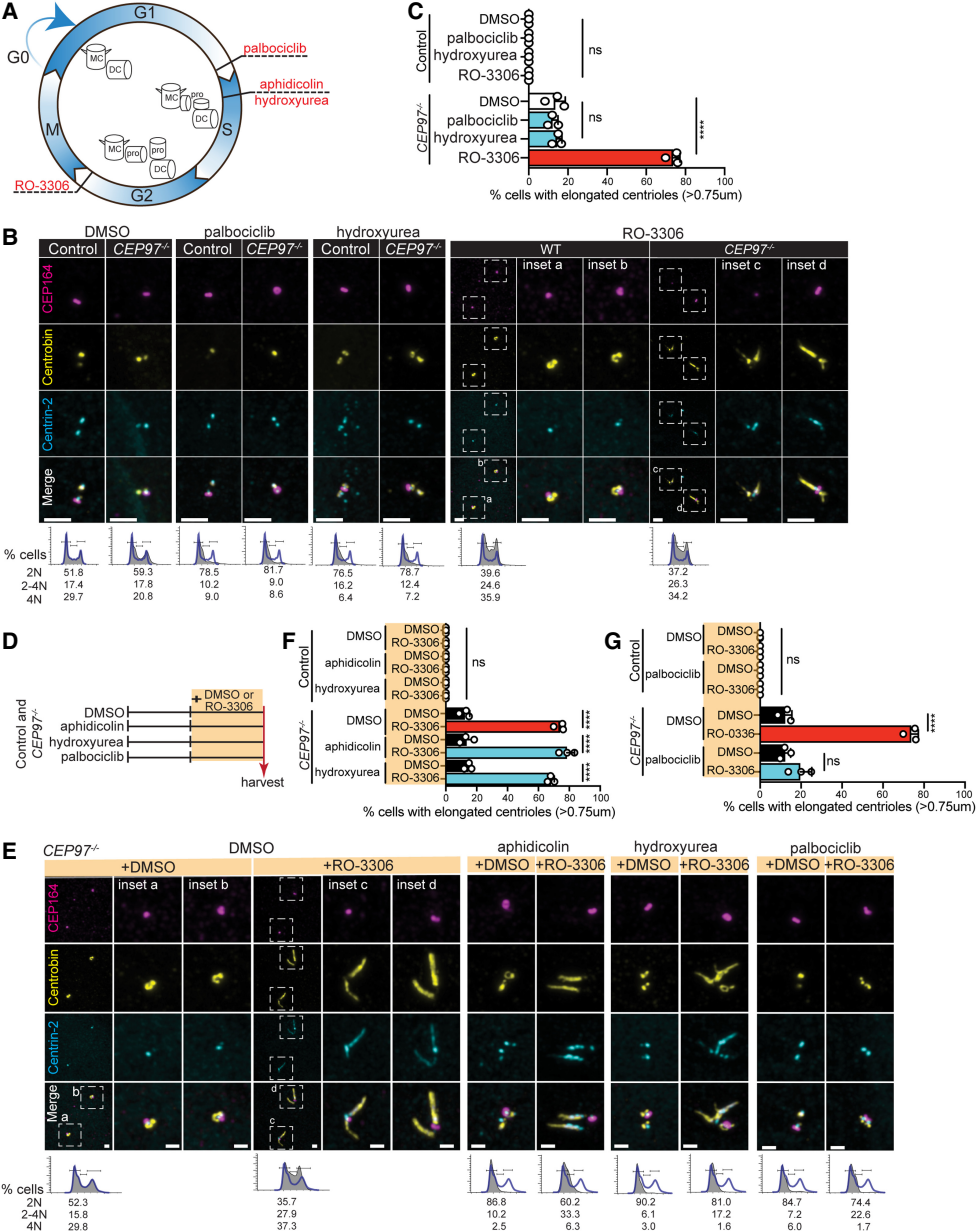
CDK1 can restrict centriole length in S phase. (*A*) A schematic of drug treatment-induced cell cycle arrest. Palbociclib induces G1 arrest, aphidicolin induces S arrest, and hydroxyurea induces S arrest. (MC) Mother centriole, (DC) daughter centriole, (pro) procentriole. (*B*, *top*) Immunofluorescence images of wild-type and *CEP97*^*–/–*^ RPE1 cells treated with DMSO, palbociclib, or hydroxyurea for 24 h and stained for CEP164 (mother centriole; magenta), Centrobin (daughter centriole predominant; yellow), and Centrin-2 (centriole; cyan). (*Bottom*) DNA content analysis of cells treated with indicated drugs. Overlaid blue traces correspond to data from the first control group. Scale bars: 2 μm for all images in *B*. (*C*) Quantification of the percentage of cells with elongated centrioles in drug-treated wild-type and *CEP97*^*–/–*^ RPE1 cells as in *B*. *n* = 3 biological replicates, with 50–100 cells per replicate. (*D*) A schematic of drug treatment of wild-type and *CEP97*^*–/–*^ RPE1. Cells were pretreated with aphidicolin, hydroxyurea, or palbociclib for 24 h, and without washout, subsequently treated with RO-3306 for 24 h before harvest. (*E*, *top*) Immunofluorescence images of *CEP97*^*–/–*^ RPE1 cells treated as in *D* and stained for CEP164 (mother centriole; magenta), Centrobin (daughter centriole predominant; yellow), and Centrin-2 (centriole; cyan). *Insets* show magnification of centrioles in boxed regions. (*Bottom*) DNA content analysis of cells treated with indicated drugs. Overlaid blue traces correspond to data from the first control group. Scale bars, 1 μm for all images in *E*. (*F*,*G*) Quantification of the percentage of cells with elongated centrioles in wild-type and *CEP97*^*–/–*^ RPE1 cells treated as in *D* and *E*. *n* = 3 biological replicates, with 50–100 cells per replicate. (*C*,*F*,*G*) Statistical significance was assessed by two-way ANOVA followed by Šídák's multiple comparison test. A *P*-value of <0.05 was considered statistically significant. (****) *P* < 0.0001. Data are represented as means ± SD.

CDK1 phosphorylates regulators of cell cycle progression as well as effectors that function independently of the cell cycle (Massacci et al. 2023). To assess whether the effects of CDK1 on centriole length are mediated via effects on cell cycle progression, we employed CDK1-independent means of triggering cell cycle arrest (Fig. 3A). CDK4/6 inhibitor palbociclib and ribonucleotide reductase inhibitor hydroxyurea inhibited cells from advancing to S phase and G2, respectively, as confirmed by measuring DNA content (Fig. 3B). Neither G1 arrest nor S-phase arrest affected centriole length in wild-type cells (Fig. 3B,C). Similarly, neither G1 arrest nor S-phase arrest exacerbated centriole overelongation in *CEP97*^*–/–*^ cells (Fig. 3B,C). Therefore, not all forms of cell cycle arrest promote centriole overelongation in the absence of CEP97.

As CDK1 activity is critical for the G2/M transition (Hochegger et al. 2008; Diril et al. 2012), we hypothesized that CDK1 inhibition promotes centriole overelongation via arrest at the G2/M transition. To test this hypothesis, we examined whether CDK1 restricts centriole length in other phases of the cell cycle. We pretreated control and *CEP97*^*–/–*^ cells with aphidicolin or hydroxyurea and then tested whether RO-3306 affected centriole length (Fig. 3D). As expected, DNA content analysis showed that aphidicolin or hydroxyurea inhibited advancement to G2, regardless of CDK1 inhibition (Fig. 3E). Furthermore, centriole number analysis indicated that aphidicolin or hydroxyurea arrested cells in S phase (Supplemental Fig. S5C). CDK1 inhibition in S-phase-arrested *CEP97*^*–/–*^ cells triggered centriole overelongation, falsifying our hypothesis (Fig. 3E,F). Instead, we conclude that CDK1 restricts centriole elongation independently of its role in mediating progression through the G2/M transition. Notably, the effect of CDK1 inhibition on promoting centriole overelongation was only observed in the absence of CEP97 (Fig. 3E,F), further indicating that CDK1 function overlaps with that of CEP97.

CDK1 is partially active in S phase but inactive in G1 (Hochegger et al. 2007; Enserink and Kolodner 2010). Therefore, we hypothesized that CDK1 inhibition in G1-arrested cells would not affect centriole length. To test this hypothesis, we arrested control and *CEP97*^*–/–*^ cells in G1 phase using palbociclib and then examined the effect of CDK1 inhibition on centriole length (Fig. 3D,E; Supplemental Fig. S5C). Consistent with our hypothesis, CDK1 inhibition had no effect on centriole length in G1-arrested cells (Fig. 3E,G). Thus, the role of CDK1 in centriole length control is cell cycle phase-specific. Taken together, our results indicate that CDK1 can restrict centriole length in S phase, independently of its canonical role in triggering progression through the G2/M transition.

### Centrobin functions downstream from CEP97 and CDK1 to regulate centriole length

If CDK1 does not act through its canonical role in cell cycle progression, how might it work with CEP97 to restrict centriole length? CAPture-phosphoMS revealed that Centrobin phosphorylation is regulated by CDK1 (Fig. 2A). Centrobin interacts with tubulin and the centriolar component CPAP and is implicated in centriole assembly, spindle assembly, microtubule polymerization, asymmetric cell division and centriole elongation (Zou et al. 2005; Jeffery et al. 2010; Gudi et al. 2011, 2015; Januschke et al. 2013; Ogungbenro et al. 2018; Balestra et al. 2021; Le Roux-Bourdieu et al. 2022; Lee et al. 2025). We hypothesized that CDK1 or CEP97 may regulate Centrobin to control centriole length.

**Figure 4.**
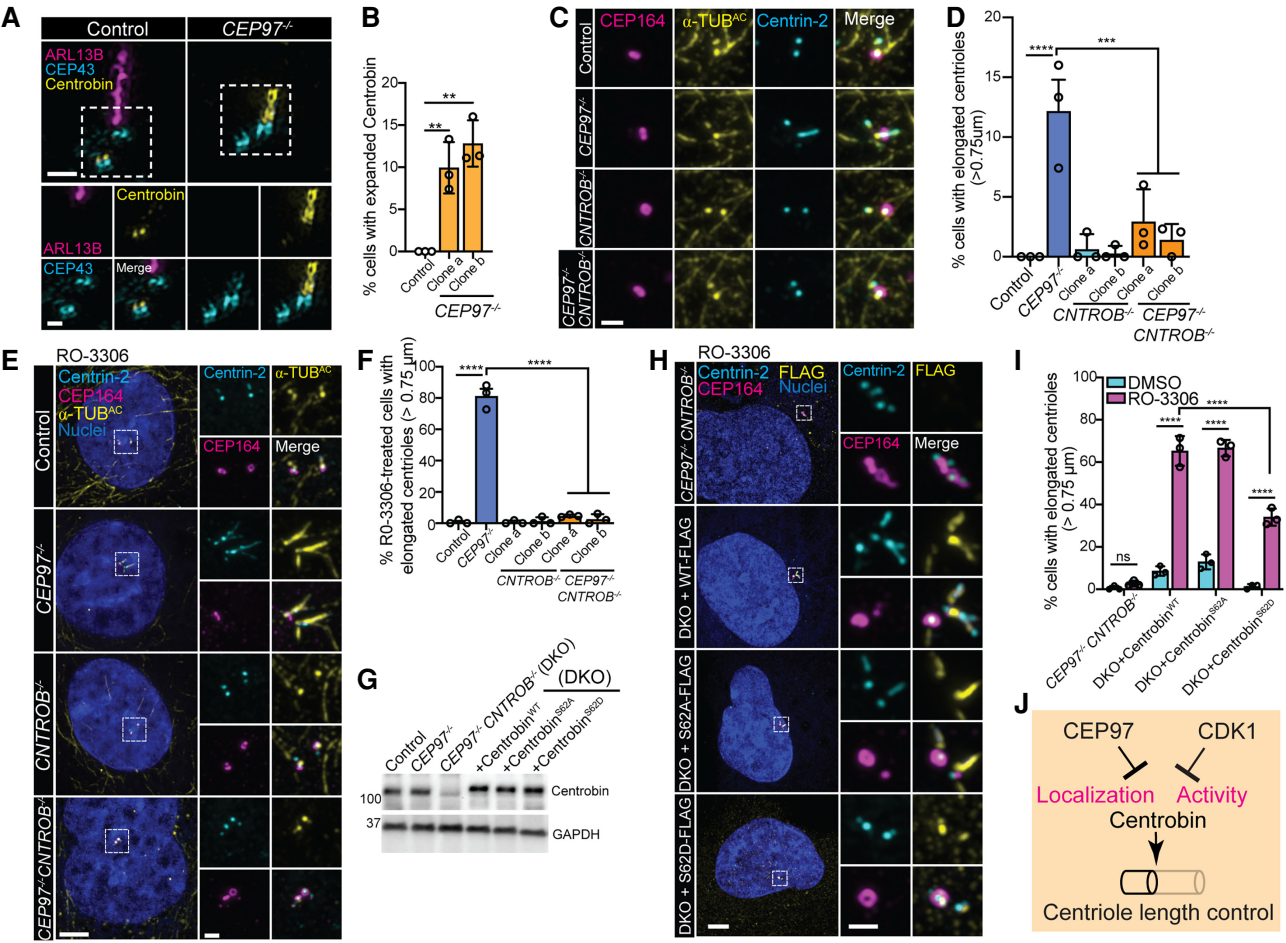
Centrobin functions downstream from CEP97 and CDK1 to regulate centriole length. (*A*) Three-dimensional structured illumination micrograph (3D-SIM) of serum starved wild-type and *CEP97*^*–/–*^ RPE1 cells stained with ARL13B (cilia, magenta), CEP43 (centriole; cyan), and Centrobin (centriole; yellow). *Inset*s show magnification of cilia and centrioles in boxed regions. Scale bars: 1 µm; *inset*, 0.5 µm. (*B*) Quantification of the percentage of cells with expanded Centrobin staining at the centrioles, as stained in *A*. *n* = 3 biological replicates, with 50–100 cells per replicate. (*C*) Immunofluorescence images of the indicated RPE1 cells stained with CEP164 (mother centriole; magenta), α-Tub^AC^ (centriole; yellow), and Centrin-2 (centriole; cyan). Scale bars, 1 µm. (*D*) Quantification of the percentage of indicated RPE1 cells with elongated centrioles, stained as in *C*. *n* = 3 biological replicates, with 50–100 cells per replicate. (*E*) Immunofluorescence images of indicated RPE1 cells treated with RO-3306 for 24 h, and stained for CEP164 (centriole; magenta), α-Tub^AC^ (centriole; yellow), Centrin-2 (centriole; cyan), and Hoechst (nuclei; blue). *Insets* show magnification of centrioles in boxed regions. Scale bars: 5 µm; *inset*, 1 µm. (*F*) Quantification of the percentage of indicated RPE1 cells with elongated centrioles after 24 h of RO-3306 treatment, as in *E*. *n* = 3 biological replicates, with 50–100 cells per replicate. (*G*) Whole-cell lysates were derived from wild-type, *CEP97*^*–/–*^, *CEP97*^*–/–*^*CNTROB*^*–/–*^ (DKO) and DKO re-expressing Centrobin^WT^, Centrobin^S62A^ or Centrobin^S62D^ and immunoblotted for indicated proteins. (*H*) Immunofluorescence images of *CEP97*^*–/–*^*CNTROB*^*–/–*^ (DKO) and DKO stably expressing FLAG-tagged Centrobin^WT^, Centrobin^S62A^, Centrobin^S62D^ RPE1 cells treated with RO-3306 for 24 h, and stained for Centrin-2 (centriole; cyan), CEP164 (mother centriole; magenta), FLAG (yellow), and Hoechst (nuclei; blue). *Insets* show magnification of centrioles in boxed regions. Scale bars: 5 µm; *inset*, 1 µm. (*I*) Quantification of the percentage of indicated RPE1 cells with elongated centrioles treated with DMSO or RO-3306 for 24 h, as in *H*. *n* = 3 biological replicates, with 50–100 cells per replicate. (*J*) Schematic model of CEP97/CDK1-Centrobin axis in centriole length control. CEP97 regulates centriole length via restriction of Centrobin localization to the centriole. CDK1-dependent phosphorylation of Centrobin S62 restricts its function. Centrobin promotes centriole growth. Statistical significance was assessed by one-way ANOVA followed by Tukey's multiple comparison tests (*B*,*D*,*F*) or two-way ANOVA followed by Šídák's multiple comparison test (*I*). A *P*-value of <0.05 was considered statistically significant. (**) *P* < 0.01, (***) *P* < 0.001, (****) *P* < 0.0001. Data are represented as means ± SD.

To begin to test this hypothesis, we examined centriolar localization of Centrobin in *CEP97*^*–/–*^ RPE1 cells. Centrobin overaccumulated on *CEP97*^*–/–*^ centrioles, suggesting that CEP97 restricts Centrobin localization at centrioles (Fig. 4A,B).

To assess whether CEP97 and Centrobin genetically interact, we generated *CEP97*^*–/–*^ *CNTROB*^*–/–*^ RPE1 cells (Supplemental Fig. S7A). Genetic ablation of Centrobin alone did not affect centriole length, consistent with previous reports (Figs. 2B,C, 4C,D; Karasu et al. 2022; Lee et al. 2025). However, deletion of Centrobin rescued centriole overelongation in *CEP97*^*–/–*^ cells, indicating that Centrobin is critical for centriole overelongation in the absence of CEP97 (Fig. 4C,D).

To further examine whether Centrobin also functions downstream from CDK1 to regulate centriole length, we examined whether centriole overelongation in RO-3306-treated *CEP97*^*–/–*^ cells depends on Centrobin. Strikingly, deletion of Centrobin prevented centriole overelongation in CDK1-inhibited *CEP97*^*–/–*^ cells (Fig. 4E,F). Re-expressing full-length FLAG-Centrobin at near-endogenous level in *CEP97*^*–/–*^ *CNTROB*^*–/–*^ cells restored centriole overelongation (Fig. 4G–I; Supplemental Fig. S7B–D). We conclude that Centrobin functions downstream from CEP97 and CDK1 to regulate centriole length.

CAPture-phosphoMS identified CDK1-dependent phosphorylation of Centrobin at S60, S62, and S790. We hypothesized that CDK1-dependent phosphorylation at these sites may inhibit the centriole elongation promoting activity of Centrobin. To test this hypothesis, we re-expressed individual phospho-dead (Centrobin-S60A, Centrobin-S62A, and Centrobin-S790A) and phospho-mimetic (Centrobin-S60D, Centrobin-S62D, and Centrobin-S790D) mutants of Centrobin at near-endogenous levels in *CEP97*^*–/–*^ *CNTROB*^*–/–*^ cells (Fig. 4G; Supplemental Fig. S7B). Centrobin^WT^, Centrobin^S60A^, Centrobin^S60D^, Centrobin^S62A^, Centrobin^S62D^, and Centrobin^S790D^ localized to centrioles whereas Centrobin^S790A^ did not (Fig. 4H; Supplemental Fig. S7E,G). In the presence of CDK1 inhibitor, Centrobin^WT^, Centrobin^S60A^, Centrobin^S60D^, Centrobin^S62A^ and Centrobin^S790D^ restored centriole overelongation (Fig. 4H,I; Supplemental Fig. S7E–H). In contrast, Centrobin^S62D^ exhibited attenuated activity (Fig. 4H,I). These results suggest that CDK1 restricts centriole length in part by regulating Centrobin phosphorylation at S62.

The attenuated activity of Centrobin^S62D^ prompted us to test whether S60 cooperates with S62 in restraining centriole elongation. We generated dual phospho-dead mutants, Centrobin^S60A/S62A^ and Centrobin^S60D/S62D^. In the presence of CDK1 inhibitor, Centrobin^S60A/S62A^, like Centrobin^S60A^, and Centrobin^S62A^, restored centriole overelongation (Supplemental Fig. S7G,H). Centrobin^S60D/S62D^, like Centrobin^S62D^, exhibited attenuated centriole elongation activity (Fig. 4H,I; Supplemental Fig.S7G,H). Together with the CDK1-dependence of Centrobin phosphorylation at S62, these findings suggest that CDK1 suppresses the centriole elongation function of Centrobin, at least in part, through phosphorylation at S62 (Fig. 4J).

### CEP97 and CDK1 cooperatively regulate centriole length in mouse embryos

**Figure 5.**
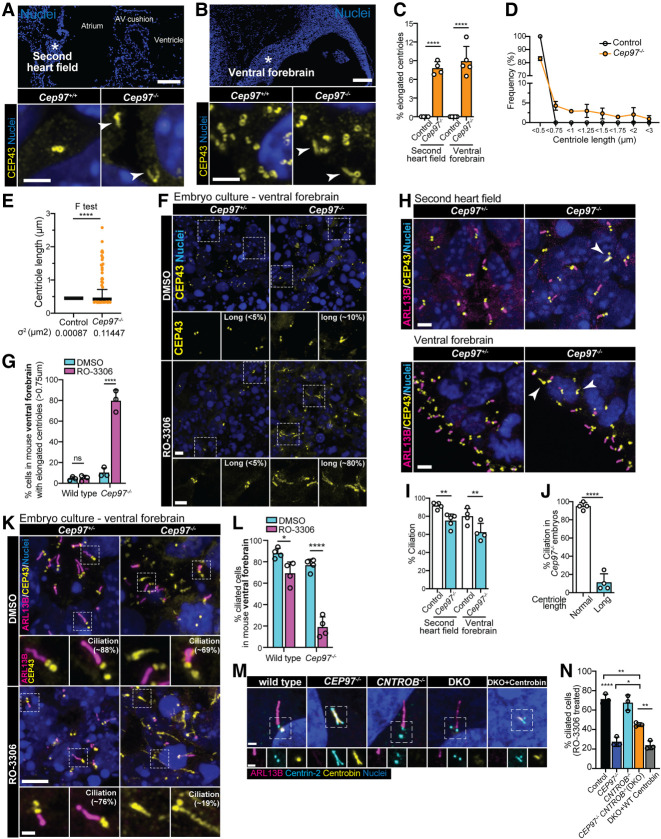
CDK1 and CEP97 regulate centriole length and promote ciliogenesis in mouse embryos. (*A*, *top* panel) Immunofluorescence images of sagittal sections of E9.5 control embryonic heart tube, stained for Hoechst (nuclei; blue). Asterisk indicates the second heart field imaged in the *bottom* panel. (*Bottom* panel) Second heart field of E9.5 *Cep97*^*–/–*^ and littermate, stained for CEP43 (centrioles; yellow) and Hoechst (nuclei, blue). Arrowheads indicate elongated centrioles. Scale bars: *top* panel, 200 µm; *bottom* panel, 2 µm. (*B*, *top* panel) Immunofluorescence images of sagittal sections of E11.5 control embryonic brain, stained for Hoechst (nuclei; blue). The asterisk indicates the ventral forebrain imaged in the *bottom* panel. (*Bottom* panel) Ventral forebrain of E11.5 *Cep97*^*–/–*^ and littermate, stained for CEP43 (centrioles; yellow) and Hoechst (nuclei; blue). Arrowheads indicate elongated centrioles. Scale bars: *top* panel, 200 µm; *bottom* panel 2 µm. (*C*) Quantification of the percentage of cells with elongated centrioles in the second heart field of E9.5 and ventral forebrain of E11.5 control and *Cep97*^*–/–*^ embryos, stained as in *A* and *B*. *n* = 4 embryos per group. (*D*) Frequency plot of centriole length from control and *Cep97*^*–/–*^ embryos, as in *A* and *B*. The *Y*-axis depicts the percentage of centrioles with length indicated on the *X*-axis. (*E*) Variance of centriole length from control and *Cep97*^*–/–*^ embryos, as in *A* and *B*. (*F*) Immunofluorescence images of ex vivo cultured E10.5 mouse embryos treated with RO-3306 for 24 h. Ventral forebrain of E10.5 *Cep97*^*–/–*^ and littermate, stained for CEP43 (centriole, yellow), and Hoechst (nuclei; blue). *Insets* show magnification of centrioles in boxed regions. Scale bars: 5 µm; *inset,* 3 µm. (*G*) Quantification of the percentage of cells with elongated centrioles in the ventral forebrain of ex vivo cultured control and *Cep97*^*–/–*^ mouse embryos treated with DMSO or RO-3306 for 24 h, as in *F*. *n* = 3 embryos per condition per group. Each data point reflects result from 50–100 cells. (*H*) Immunofluorescence images of second heart field (*top* panel) of E9.5 *Cep97*^*–/–*^ and littermate and ventral forebrain (*bottom* panel) of E11.5 *Cep97*^*–/–*^ and littermate, stained for ARL13B (cilia; magenta), CEP43 (centrioles, yellow) and Hoechst (nuclei, blue). Arrowheads indicate elongated centrioles. Scale bars, 3 µm. (*I*) Quantification of ciliation percentage in the second heart field of E9.5 and forebrain of E11.5 wild-type and *Cep97*^*–/–*^ embryos, stained as in *H*. *n* = 4 embryos per group. (*J*) Quantification of cilia associated with normal and long centrioles in the second heart field of E9.5 *Cep97*^*–/–*^ embryos, stained as in *H*. *n* = 4 embryos per group. (*K*) Immunofluorescence images of ex vivo cultured E10.5 mouse embryos treated with DMSO or RO-3306 for 24 h. Ventral forebrain of E10.5 *Cep97*^*–/–*^ and littermate, stained for ARL13B (cilia; magenta), CEP43 (centriole; yellow), and Hoechst (nuclei; blue). *Inset*s show magnification of cilia and centrioles in boxed regions. Scale bars: 5 µm; *inset*, 1 µm. (*L*) Quantification of the percentage ciliated cells in the ventral forebrain of ex vivo cultured wild-type and *Cep97*^*–/–*^ mouse embryos treated with DMSO or RO-3306 for 24 h, as in *K*. (*M*) Immunofluorescence images of indicated RPE1 cells treated with RO-3306 for 24 h, and after washout, cultured in serum-free medium for 48 h, and stained for ARL13B (cilia, magenta), Centrin-2 (centriole, cyan), Centrobin (centriole, yellow), and Hoechst (nuclei, blue). *Insets* show magnification of cilia and centrioles in boxed regions. Scale bars: 1 μm; *insets*, 1 μm. (*N*) Quantification of the percentage of ciliated RPE1 cells treated as in *M*. *n* = 3 biological replicates, with 50–100 cells per replicate. Statistical significance was assessed by Student's *t*-test (*J*), by one-way ANOVA followed by Tukey's multiple comparison tests (*N*), by two-way ANOVA followed by Šídák's multiple comparison test (*C*,*G*,*I*,*L*), or by *F*-test of equal variance (*E*). A *P*-value of <0.05 was considered statistically significant. (*) *P* < 0.05, (**) *P* < 0.01, (****) *P* < 0.0001. Data are represented as means ± SD.

To determine whether CEP97 restricts centriole length in vivo, we examined the phenotype of *Cep97*^*–/–*^ mice. *Cep97*^*–/–*^ mice died at birth, exhibiting cyanosis and preaxial polydactyly (Supplemental Fig. S8A,B). Given the established roles of centrosomal and ciliary genes in heart and brain development (Li et al. 2015; Bouman et al. 2017; Jin et al. 2017; Snedeker et al. 2017; Jayaraman et al. 2018; Watkins et al. 2019; Yang et al. 2021; Duy et al. 2024; Wang et al. 2025), we examined centriole morphology in cardiac and neural progenitors. Eight percent of *Cep97*^*–/–*^ E9.5 second heart field cells contained abnormally long centrioles (Fig. 5A,C). Similarly, 9% of *Cep97*^*–/–*^ E11.5 ventral and dorsal forebrain cells contained abnormally long centrioles (Fig. 5B,C; Supplemental Fig. S8C–E). Control second heart field and forebrain cell centrioles exhibited low variance in centriole length (Fig. 5D,E). In contrast, *Cep97*^*–/–*^ second heart field and forebrain cell centrioles exhibited high variance in centriole length (Fig. 5D,E).

Interestingly, centriole overelongation in *Cep97*^*–/–*^ embryos was tissue-specific. For example, in the caudal neural tube, a tissue in which centrioles and cilia are critical for development (Huangfu et al. 2003; Bazzi and Anderson 2014), removing CEP97 did not lead to centriole overelongation (Supplemental Fig. S8F). Thus, during mouse development, CEP97 is required for centriole length control in heart and brain anlagen.

To assess the role of CDK1 on centriole length during mouse development, we treated cultured E10.5 control and *Cep97*^*–/–*^ mouse embryos with RO-3306. RO-3306 increased punctate nuclear pHH3 staining, a marker of late G2 phase (Hendzel et al. 1997; Contestabile et al. 2009; Acin et al. 2011), indicating that CDK1 activity was inhibited in cultured mouse embryos (Supplemental Fig. S9A–D). Inhibiting CDK1 did not affect centriole length in control embryos (Fig. 5F,G). In contrast, CDK1 inhibition increased the proportion of cells with elongated centrioles in *Cep97*^*–/–*^ embryos eightfold (Fig. 5F,G). Thus, similar to RPE1 cells, mouse embryos rely on CEP97 and CDK1 to act together to restrict centriole elongation.

### CDK1 and CEP97 cooperatively promote ciliogenesis in mouse embryos

Centrioles nucleate primary cilia and are critical for heart and brain development (Jayaraman et al. 2018; Djenoune et al. 2022; Mill et al. 2023). As CEP97 helped restrict centriole length in the second heart field and forebrain, we investigated whether CEP97 supported ciliogenesis during mouse development. Indeed, *Cep97*^*–/–*^ embryos exhibited reduced ciliation in forebrain neuronal progenitor cells and in second heart field cardiac progenitor cells (Fig. 5H,I). In contrast, *Cep97*^*–/–*^ embryos exhibited normal ciliation in the developing spinal cord, a tissue where loss of CEP97 did not alter centriole morphology (Supplemental Fig. S8F). Within the *Cep97*^*–/–*^ second heart field and forebrain, abnormally long centrioles correlated with compromised ciliogenesis (Fig. 5J). These results suggest that CEP97 promotes ciliogenesis via centriole length regulation in select tissues.

To assess whether CDK1 also promotes ciliogenesis in embryos, we examined cilia formation in mouse embryos treated with CDK1 inhibitor. CDK1 inhibition did not dramatically reduce ciliogenesis in wild-type embryos but did in *Cep97*^*–/–*^ embryos (Fig. 5K,L). These results are consistent with the conclusion that CEP97 and CDK1 function together in mammalian development to control centriole length, critical for ciliogenesis.

Centriole length is restored to wild-type levels in *CEP97*^*–/–*^ *CNTROB*^*–/–*^ RPE1 cells, compared to *CEP97*^*–/–*^ cells (Fig. 4C–F), raising the possibility that ciliogenesis may also be restored. As with cultured mouse embryos, CDK1 inhibition dramatically attenuated ciliogenesis in *CEP97*^*–/–*^ RPE1 cells, while only mildly affecting ciliogenesis in control cells (Supplemental Fig. S10A,B). Remarkably, removal of Centrobin partially restored ciliogenesis in CDK1-inhibited *CEP97*^*–/–*^ cells (Fig. 5m,N). Moreover, re-expression of wild-type Centrobin at near-endogenous levels in CDK1-inhibited *CEP97*^*–/–*^ *CNTROB*^*–/–*^ cells restored centriole overelongation and disrupted ciliogenesis, similar to *CEP97*^*–/–*^ cells (Fig. 5m,N). We conclude that CDK1 and CEP97 promote ciliogenesis via restricting centriole length.

### CEP97 promotes mitotic progression in brain development

Because centrosomes contribute to the mitotic spindle and promote progression through mitosis during brain development (Phan and Holland 2021; Stracker 2024; Wong et al. 2025), we hypothesized that CEP97 may, in addition to promoting ciliogenesis, facilitate mitotic progression. To test this hypothesis, we quantified the proportion of mitotic cells in the embryonic brain. The proportion of neural progenitor cells in mitosis was increased approximately fourfold and threefold in the dorsal and ventral telencephalons of *Cep97*^*–/–*^ embryos, respectively (Supplemental Fig. S11A,B). Examination of the mitotic figures revealed that *Cep97*^*–/–*^ embryos also exhibited increased mitoses with monopolar spindles (Supplemental Fig. S11C,D). Thus, CEP97 is important for bipolar spindle formation and mitotic progression in neural progenitors.

### CEP97 is required for HH signaling in heart development

**Figure 6.**
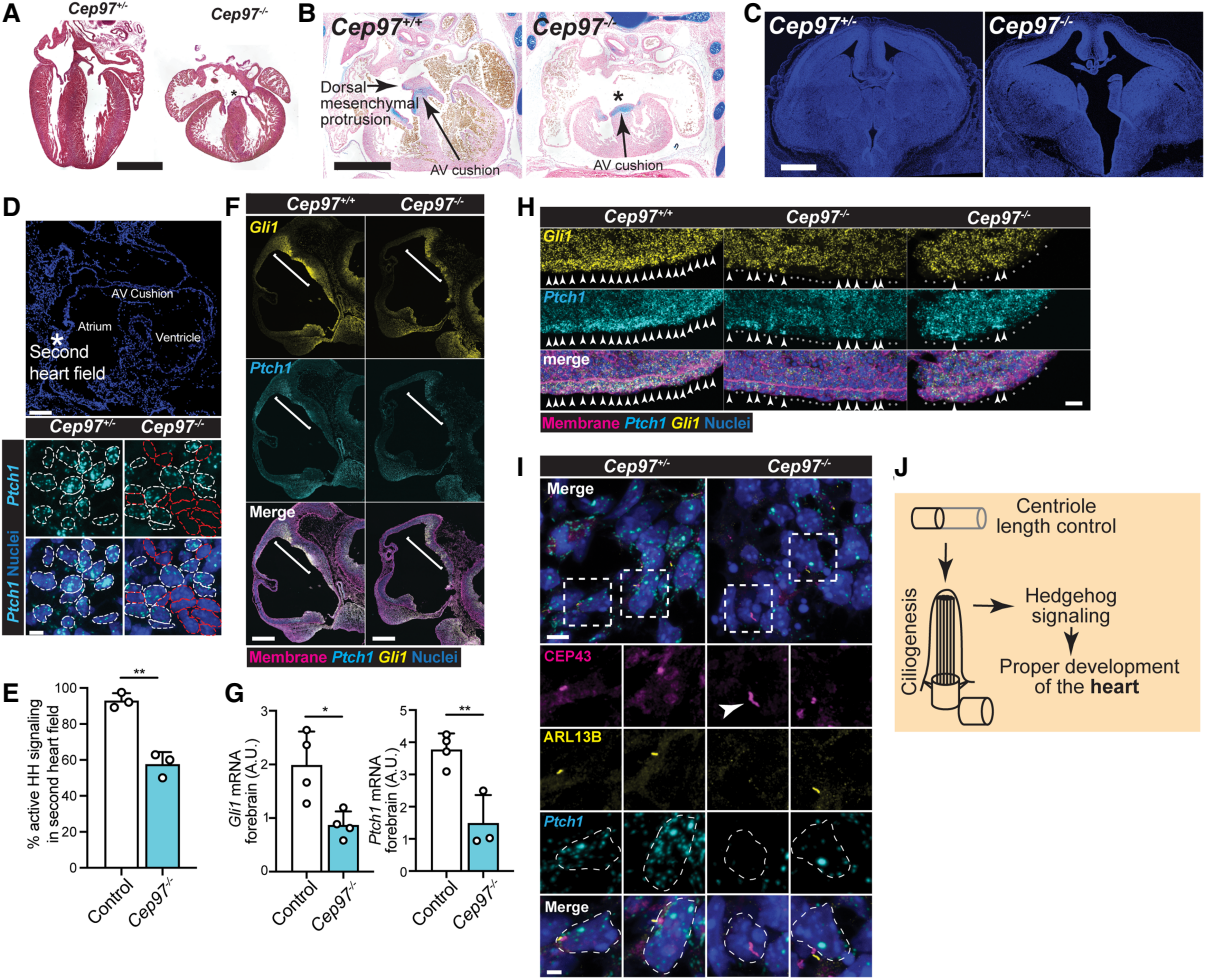
CEP97 regulates HH signaling and brain and heart development in mice. (*A*) Histology of *Cep97*^*–/–*^ and littermate control mouse hearts at P0. Asterisks indicate atrio–ventricular septal defect. Scale bar, 1 mm. (*B*) Alcian blue stain of E15.5 wild-type and *Cep97*^*–/–*^ embryos, counter stained with nuclear fast red. The dorsal mesenchymal protrusion is absent in the *Cep97*^*–/–*^ embryo (asterisk). Scale bar, 500 µm. (*C*) Hoechst (nuclei, blue) staining of E14.5 control and *Cep97*^*–/–*^ embryonic frontal plane of the head. Scale bars, 500 µm. (*D*, *top* panel) Immunofluorescence images of sagittal sections of E9.5 control embryonic heart tube, stained for Hoechst (nuclei; blue). The asterisk indicates the second heart field. (*Bottom* panel) Transcripts of HH signaling target genes *Ptch1* (cyan), assayed by RNAscope, in the second heart field of *Cep97*^*–/–*^ and littermate control mouse embryos at E9.5, and stained for Hoechst (nuclei; blue). Cells were considered as HH signaling active if presented with two or more *Ptch1* mRNA fluorescence dots. The nuclei of cells in active/inactive HH signaling state were outlined with dotted white/red lines, respectively. Scale bars: 100 µm; *inset*, 5 µm. (*E*) Quantification of the percentage of cells in the second heart field of wild-type and *Cep97*^*–/–*^ mouse embryos at E9.5 that were in an active HH signaling state, as in *D*. *n* = 3 embryos per genotype. (*F*) Transcripts of HH signaling target genes, *Gli1* and *Ptch1*, assayed by RNAscope, in the brains of *Cep97*^*–/–*^ and littermate control embryos at E11.5, and stained for Hoechst (nuclei, blue) and membrane (Wheat Germ Agglutinin, magenta). Scale bars, 400 µm. (*G*) Quantification of fluorescence intensities of *Gli1* and *Ptch1* transcripts in the brains of wild-type and *Cep97*^*–/–*^ mouse embryos at E11.5, as in *F*. *n* = 3–4 embryos per genotype. (*H*) Transcripts of HH signaling target genes *Gli1* and *Ptch1*, assayed by RNAscope, in the zone of polarizing activity of the hindlimb of *Cep97*^*–/–*^ and littermate control mouse embryos at E11.5, and stained for Hoechst (nuclei; blue) and wheat germ agglutinin (membrane; magenta). Arrowheads indicate cells exhibiting active HH signaling. Asterisks indicate cells exhibiting inactive HH signaling. Scale bars, 20 µm. (*I*) Transcripts of HH signaling target gene *Ptch1*, assayed by RNAscope, in the second heart field of *Cep97*^*–/–*^ and littermate control mouse embryos at E9.5, and stained for CEP43 (centriole; magenta), ARL13B (cilia; yellow), and Hoechst (nuclei; blue). Scale bars: 5 µm; *inset*, 2 µm. (*J*) Schematic illustrating that centriole length control impacts ciliogenesis to support HH signal transduction and developmental patterning in the heart. Statistical significance was assessed using a two-tailed unpaired *t*-test in *E*,*G*. A *P*-value of <0.05 was considered statistically significant. (*) *P* < 0.05, (**) *P* < 0.01. Data are represented as means ± SD.

Cilia and centrosomes are critical for heart and brain development (Li et al. 2015; Bouman et al. 2017; Jin et al. 2017; Jayaraman et al. 2018; Watkins et al. 2019; Duy et al. 2024; Wang et al. 2025). Consistently, *Cep97*^*–/–*^ embryos developed atrio–ventricular septal defect (AVSD) and microcephalic ventriculomegaly, observed both by micro-computed tomography (µCT) and histology (Fig. 6A–C; Supplemental Fig. S12A–C).

The second heart field gives rise to the dorsal mesenchymal protrusion, which together with endothelial-derived atrio–ventricular cushion contribute to cardiac septation (Supplemental Fig. S12D,E; Burns et al. 2016). The atrio–ventricular cushion was present in E15.5 *Cep97*^*–/–*^ embryos and in P0 *Cep97*^*–/–*^ hearts (Fig. 6B; Supplemental Fig. S12F). In contrast, dorsal mesenchymal protrusions were absent in Cep97^−/−^ hearts (Fig. 6B; Supplemental Fig. S12F). Thus, CEP97 is critical for dorsal mesenchymal protrusion formation, involved in atrio–ventricular septation.

Centrosomes and primary cilia are important for vertebrate HH signaling (Huangfu et al. 2003; Corbit et al. 2005; Rohatgi et al. 2007; Truong et al. 2021). HH signaling helps pattern the developing heart, neural tube and limb bud (Dessaud et al. 2008; Goddeeris et al. 2008; Komada et al. 2008; Briggs et al. 2016). HH signaling activates transcription of genes such as *Gli1* and *Ptch1*. Single-molecule in situ hybridization of *Cep97*^*–/–*^ embryos for transcripts of *Gli1* and *Ptch1* revealed that CEP97 was critical for HH signal transduction in the second heart field, forebrain and limb buds (Fig. 6D–H; Supplemental Fig. S13A). In contrast, in the developing spinal cord, a tissue in which CEP97 was dispensable for ciliogenesis, CEP97 was also dispensable for HH signal transduction and developmental patterning (Supplemental Fig. S13B–D). Therefore, CEP97 is important for HH-mediated development in select tissues (e.g., heart and brain) in which CEP97 is required for ciliogenesis.

Close inspection of *Gli1* and *Ptch1* expression revealed that, even within the same *Cep97*^*–/–*^ tissue, HH-responsive and nonresponsive cells were intermingled (Fig. 6D; Supplemental Fig. S13A). As we had found that centriole overelongation in *CEP97*^*–/–*^ RPE1 cells and in *Cep97*^*–/–*^ embryos was nonuniform (Figs. 2F–I, 5A–E), we hypothesized that the *Cep97*^*–/–*^ cells possessing overelongated centrioles may also be the cells with abrogated HH responsiveness. To test this hypothesis, we combined immunofluorescent staining of centriole and cilia with single-molecule in situ hybridization for HH target gene *Ptch1*.

In the control *Cep97*^*+/–*^ second heart field, cardiac progenitors uniformly contained normal centrioles, possessed cilia, and expressed *Ptch1*, indicative of active HH signaling (Fig. 6I). In contrast, in the *Cep97*^*–/–*^ second heart field, cardiac progenitors containing abnormally long centrioles lacked cilia and showed abrogated HH signal transduction, whereas adjacent cells containing normal centrioles possessed cilia and showed active HH signal transduction (Fig. 6I).

Taken together, we conclude that CEP97 restricts centriole length to promote ciliogenesis, and that stochastic defects in ciliogenesis disrupt HH signal transduction and developmental patterning in the heart (Fig. 6J).

## Discussion

Protein phosphorylation orchestrates cell cycle progression and organelle biogenesis. CDK1, a master regulator of the cell cycle, also helps govern organelle biogenesis (Taguchi et al. 2007; Holt et al. 2009; Shiota et al. 2015; Massacci et al. 2023). During centrosome biogenesis, CDK1 regulates number and maturation, but few centrosomal targets of CDK1 have been identified (Okuda et al. 2000; Fisk and Winey 2001; Chen et al. 2002; Fisk et al. 2003; Hochegger et al. 2007; Holt et al. 2009; Wang et al. 2014; Novak et al. 2016; Zitouni et al. 2016; Huang et al. 2022; Steinacker et al. 2022; Roberts et al. 2024). We developed a centrosome-specific phosphoproteomics approach, CAPture-phosphoMS and identified that centrosomal proteins, such as Centrobin, are phosphorylated in a CDK1-dependent way. CDK1 mediates phosphorylation of Centrobin at serine 62, inhibiting the ability of Centrobin to promote centriole elongation.

CEP97-CCP110 is a distal centriolar protein complex that also inhibits centriole elongation, but by inhibiting the accumulation of Centrobin at centrioles. Both in cultured human cells and in developing mouse embryos, CDK1 and CEP97-CCP110 synergistically restrict centriole elongation. This cooperative control of centriole length acts via inhibiting Centrobin via two different mechanisms: a negative regulatory phosphorylation and control of subcellular localization.

Despite both working through Centrobin, CDK1 and CEP97-CCP110 likely impart different forms of regulation over centriole elongation. CDK1 is well recognized as a global orchestrator of cellular functions, exhibiting oscillatory activity attuned to the cell cycle, whereas CEP97-CCP110 acts locally at the distal centriole (Iyer et al. 2025). It will be interesting to determine whether for organelles other than the centrosome, CDK1 exerts organelle-specific functions by working in coordination with local factors.

It is important to distinguish that while CDK1 promotes key aspects of cell cycle progression (e.g., DNA synthesis, G2/M transition and spindle assembly), it is itself regulated by cell cycle factors such as WEE1, PKMYT1 and CDC25C. We found that CDK1 activity restrains centriole length in S phase, before CDK1 activity is required for cell cycle progression. Thus, the effects of CDK1 on centriole length control are not secondary to effects on cell cycle progression. Still, CDK1 activity is coordinated with cell cycle events, including centriole biogenesis, and CDK1 activity is dispensable for centriole length control in G1. Thus, the temporally regulated CDK1 activity is relevant to when CDK1 functions in centriole elongation regulation and may allow CDK1 to coordinate centrosome growth with overall cell growth (Aydogan et al. 2020).

Centriole length regulation by CDK1 and CEP97 is critical to support ciliogenesis in human cells and during mouse embryonic development. Without CEP97, dysregulated centriole length and impaired ciliogenesis interrupt HH signal transduction, disrupting heart development. Other centriolar and ciliary proteins implicated in mammalian heart development (e.g., TBC1D32, MKS1, NEK8) may likewise be important for ciliogenesis and HH signal transduction in human heart progenitors (Li et al. 2015; Zaidi and Brueckner 2017). Given the cooperative functions of CEP97-CCP110 and CDK1, it will be interesting to understand whether increased CDK1 inhibition, like that imparted by WEE1, exacerbates centriole- and cilia-related birth defects. CEP97 also contributes to brain development, possibly through its role in spindle formation and mitotic progression.

The partner of CEP97, CCP110, binds directly to microtubules and antagonizes the function of CPAP (Schmidt et al. 2009; Iyer et al. 2025). As Centrobin binds to CPAP and promotes its centriolar localization (Gudi et al. 2015), it is likely that CEP97 antagonizes the function of both Centrobin and CPAP. The Centrobin phospho-mimetic mutants generated in this study did not prevent all centriole overelongation that occurs in the absence of CEP97 and CDK1 activity, indicating that CDK1 is likely to restrict centriole length via additional Centrobin phosphorylation events or other factors. Other factors that may help CDK1 restrict centriole elongation include CEP135 and CEP295, both of which have been previously implicated in centriole elongation and exhibit CDK1-dependent phosphorylation (Supplemental Table S5; Lin et al. 2013; Chang et al. 2016).

Previous work found that CDK1 controls centriole number via phosphorylation of STIL (Zitouni et al. 2016; Steinacker et al. 2022), and regulates the localization of CEP152 and PCNT in oocytes (Lee et al. 2018). Thus, CDK1 controls multiple aspects of centrosome function beyond centriole elongation. Apart from Centrobin, STIL, CEP152 and PCNT, CAPture-phosphoMS identified numerous centriolar proteins phosphorylated in a CDK1-dependent way. Other centriolar components that may be phosphorylated by CDK1 include proteins involved in centriole duplication (e.g., CEP192), centriole cohesion (e.g., CEP68), mother centriole maturation (e.g., OFD1) and microcephaly (e.g., CEP135, NIN) (Graser et al. 2007; Wang et al. 2009; Singla et al. 2010; Lee and Rhee 2011; Dauber et al. 2012; Fang et al. 2014; Fu et al. 2016; Lee et al. 2018; Kumar et al. 2021). Thus, it is likely that CDK1, in a way that may be partially overlapping with CDK2, phosphorylates many centrosomal proteins to shape multiple aspects of centrosomal function.

Outside of the centrosome, some cellular structures are robust to even drastic morphological changes. For example, the mitotic spindle can execute chromosome segregation despite changes in its size and the ER can traffic proteins despite changes in its shape (Wilbur and Heald 2013; Mukherjee and Levy 2019). In contrast, the size and shape of centrioles are key to their functions and, consequently, centriole geometry appears to be more rigidly constrained. The regulatory mechanisms identified for centrioles may illustrate principles, such as overlapping function between organelle-integral factors (e.g., CEP97-CCP110) and dynamic cell cycle regulators (e.g., CDK1) ensuring that the regulation of distinct organelles is harmonized both with each other and with cell growth.

## Materials and methods

### Mouse lines

*Cep97*^+/*–*^ [*Cep97*^tm1(KOMP)Vlcg^] mice, generated in a C57BL/6NJ background, were obtained from the International Mouse Phenotyping Consortium (IMPC) and backcrossed to C57BL/6J mice for 10 generations to eliminate background mutations. In all animal assays conducted in this study, *Cep97*^*+/+*^ and *Cep97*^*+/–*^ mice were phenotypically indistinguishable and were used as littermate controls. *mef2c*-AHF-Cre; Rosa26YFP [*Gt(ROSA)26Sortm1(EYFP)Cos*] mice were described previously (Verzi et al. 2005; Kopinke et al. 2017).

Mice were housed in a barrier facility with veterinary supervision and given food and water ad libitum. All mouse protocols were approved by the Institutional Animal Care and Use Committee at the University of California, San Francisco.

### Mammalian cell culture

Human retinal epithelial (RPE1-hTERT) cells were cultured in DMEM/F12 (University of California, San Francisco, Media Production: CCFAA010) supplemented with 10% FBS and 1× GlutaMAX at 37°C and 5% CO_2_. Lenti-X 293T cells were cultured in DMEM (University of California, San Francisco Media Production: CCFAA005) supplemented with 10% FBS and 1× GlutaMAX at 37°C and 5% CO_2_. All cells were routinely checked for mycoplasma infection and were negative for mycoplasma infection.

### Ex vivo embryo culture

Ex vivo embryos were cultured as described previously (Rojas et al. 2005). Briefly, mouse embryos from *Cep97*^+/*–*^ and *Cep97*^+/*–*^ breeding pairs were harvested at 10.5 dpc, and embryonic tissues cranial to the septum transversum were dissected and cultured in Dulbecco's modified Eagle's medium supplemented with 1% fetal bovine serum, 100 U/mL penicillin, 100 U/mL streptomycin, and 2 mM L-glutamine at 37°C and 5% CO_2_ in 24 well tissue culture plates. RO-3306 (50 µM) (Sigma-Aldrich SML0569) or DMSO was added to the cultured embryonic tissue.

### Generation of knockout RPE1 cells by CRISPR–Cas9

Cycling RPE1 cells were trypsinized, counted, washed in PBS, and resuspended in SG electroporation buffer (Lonza V4SC-3096) at a concentration of 10,000 cells per 1 µL. Guide RNAs targeting *CEP97, CCP110, OFD1*, and *Centrobin* were designed and synthesized (Synthego) (sequences in Supplemental Table S6). RNPs were assembled by incubating 45 pmol of TrueCut Cas9 v2 protein (Thermo Fisher A36499) with 90 pmol of sgRNA for 15 min at room temperature. A total of 200,000 cells in 20 µL of SG buffer were added to each RNP and electroporated using program 96-EA-104 on an Amaxa 4D nucleofector equipped with a 96 well shuttle (Lonza AAF-1003B/S). After electroporation, cells were collected in complete culture medium and seeded onto 6 well plates. Three days later, the monoclonal cell line was further selected by seeding cells in 96 well plates at 0.3 cell per well. Clonal cell lines were expanded and genotyped. To verify cell line genotype, gDNA was extracted from a clonal cell line using an AllPrep DNA/RNA mini kit (Qiagen 0204). gDNA was amplified using oligonucleotides surrounding the sgRNA binding site. PCR amplicons were purified and Sanger-sequenced using the corresponding forward primers. In addition, PCR-verified knockout clones were further validated by either Western blot or immunofluorescence staining.

### Generation of stable cell lines

The pLVX-Neo-Actn1-GFP plasmid was obtained from Addgene (138293). For expression of CEP97 or Centrobin, human CEP97 or Centrobin was PCR-amplified. CEP97 was cloned into pLVX-Neo-Actn1-GFP with the promoter replaced with EF1α-ΔTATA, and Centrobin was cloned under the control of a Tet-On promoter. Centrobin mutant clones were constructed with In-Fusion Snap Assembly (Takara 638948).

For lentiviral production, 5 µg of pLVX, 1.4 µg of pMD2.G (Addgene 12259), and 3.9 µg of psPAX2 (Addgene 12260) were transfected into a 10 cm plate of Lenti-X 293T cells (HEK 293T; Takara) at 70%–80% confluence using Fugene 6 transfection reagent (Promega E2691). Medium containing lentiviral particles was filtered with a 0.45 µm PES membrane filter (Genesee Scientific 25-246) and concentrated with Lenti-X concentrator (Takara 631232) according to the manufacturer's instructions. Lentiviral particles were resuspended in 100 µL of PBS, aliquoted, and snap-frozen at −80°C.

For generation of stable cell lines, RPE1 cells were transduced with 10 µL of lentiviral particles in a 6 well plate at 50% confluence. After 24 h, cells were changed to fresh medium containing 10 µg/mL puromycin or 1000 µg/mL G418. Puromycin was removed when live cells were no longer observed in the nontransduced control condition.

### Centriole elongation assay in response to drug treatment

For cultured RPE1 cells, centriole elongation assay was conducted as described previously (Peneda et al. 2020). Briefly, asynchronous RPE1 cells were treated with drugs for 24 h before harvesting for downstream immunofluorescence or cell cycle analysis. Drugs were used at the following concentrations: 1 µM K03861 (Selleckchem S8100), 150 nM palbociclib (Selleckchem S1579), 4 µM aphidicolin (Sigma A0781), 4 mM hydroxyurea (Sigma H8627), 10 µM RO-3306 (Sigma SML0569), and 100 µM etoposide (Fisher 33419-42-0).

### Immunofluorescence of RPE1 cells

Cells were washed twice with PBS before being fixed with either freshly prepared 4% paraformaldehyde (PFA) for 10 min at room temperature or methanol for 5 min at −20°C. Cells were blocked for 1 h at room temperature in PBT (1% BSA, 0.5% Triton X-100, 0.02% sodium azide in PBS). Cells were then incubated in primary antibody diluted in PBT overnight at 4°C. After three washes in PBST (0.1% Tween 20 in PBS) for 10 min each, secondary antibodies and Hoechst diluted in PBT were added for 1 h at room temperature. Secondary antibodies and Hoechst were washed off as done for the primary antibody. Cells were then mounted in Prolong Diamond antifade medium (Molecular Probes P36970). For tertiary staining, cells were further blocked after secondary and Hoechst wash off with rabbit IgG (Cell Signaling Technology 3900S) for 1 h at room temperature. Cells were then incubated with Alexa Fluor555-conjugated tertiary antibody overnight at 4°C. After three washes in PBST for 10 min each, cells were then mounted in Prolong Diamond antifade medium (Molecular Probes P36970).

### Tissue section, immunofluorescence, and histology

Tissues from ex vivo cultured mouse embryos, mouse embryos, or P0 mice were dissected and fixed in 4% PFA overnight at 4°C, washed three times in PBS, and placed into 15% sucrose and 30% sucrose until tissues descended. Tissues were embedded in OCT and sectioned on a Leica CM1900 cryostat at 10 µm thickness. Sections were collected on glass slides and allowed to dry for at least 1 h before staining. A hydrophobic pen was used to encircle tissues. For staining, sections were washed three times in PBST (0.1% Tween-20 diluted in PBS) and blocked in 1% BSA and 0.4% M.O.M (Vector Laboratories MKB-2213-1) in PBST for 1 h at room temperature. Primary antibodies were diluted in PBST and 1% BSA and placed on slides overnight at 4°C. Slides were washed three times in PBST, and secondary antibody was added for 1 h at room temperature. Slides were washed three times for 5–10 min each in PBST and mounted with Prolong Diamond antifade medium. Primary and secondary antibodies, tertiary stains, dilutions, and fixation conditions are described in Supplemental Table S7.

For histological analysis, mouse embryos at E15.5 were harvested and dissected below the septum transversum, and embryonic tissues cranial to the septum transversum were dissected and fixed in 4% PFA overnight at 4°C. P0 mice were euthanized and the hearts were dissected, washed three times in PBS, and fixed in 4% PFA overnight at 4°C. Fixed tissues were embedded in paraffin, and 5 µm sections were cut and processed for hematoxylin and eosin (H&E) staining or Alcian blue staining.

### In situ hybridization

Single-molecule in situ hybridization was performed using Advanced Cell Diagnostics RNAscope 2.0 HD detection kit for the following probes: Mm*-Ptch1* (402811) and Mm-*Gli1* (311001).

### Image acquisition

Airyscan and confocal images of immunofluorescent stainings were acquired on a Zeiss LSM 800 laser scanning confocal microscope equipped with a 63×/1.4 NA oil immersion objective and captured using the Zen imaging software (Zeiss). Superresolution 3D-SIM images were acquired using a DeltaVision OMX-SR microscope (GE Healthcare) using a 60×/1.42 NA oil immersion objective and three scientific complementary metal-oxide semiconductor cameras. Immersion oil with a refractive index of 1.518 was used. *Z*-stacks of 6 µm were collected using a 0.125 µm step size. Raw images were reconstructed using SoftWoRx 6.5.2 (GE Healthcare) using default parameters. Images were processed using Fiji software to generate maximal projections and quantifications.

H&E staining was acquired on a Nikon Ti inverted microscope equipped with a plan apo 10×/0.45 NA objective and captured using a Nikon DS-Ri2 color camera.

### Micro-CT

E17.5 mouse embryos were harvested and fixed overnight in 4% PFA at 4°C and subjected to micro-CT (µCT) at the Small Animal Imaging Shared Facility at the University of Alabama at Birmingham. Briefly, PFA-fixed embryos were stabilized with hydrogel and infiltrate with isotonic aqueous potassium triiodide (Lugol solution). The embryos were imaged at 8 µm resolution using a Scanco Medical µCT40 instrument. The embryos were scanned at 70 kVp and 114 µA with an integration time of 300 msec and with 1000 projections per 180°. µCT data were processed with 3D Slicer (v.3.8.1) to generate heart and brain images.

### Immunoblotting

Cells were lysed using SDS lysis buffer (10 mM Tris at pH 7.4, 100 mM NaCl, 1 mM EDTA, 1 mM EGTA, 1 mM NaF, 20 mM Na_4_P_2_O_7_, 2 mM Na_3_VO_4_, 1% Triton X-100, 10% glycerol, 0.1% SDS, 0.5% deoxycholate) (Invitrogen FNN0011) and protease and phosphatase inhibitors (Thermo Scientific A32961). Protein concentration was determined using a Pierce BCA protein assay kit (Thermo Fisher Scientific). All lysates were boiled for 5 min in 4× LDS sample buffer (Invitrogen NP0008). Protein samples were separated on 4%–15% gradient TGX precast gels (Bio-Rad) and transferred to PVDF membrane (Bio-Rad). Two percent BSA in TBS with 0.1% Tween was used to block membranes and dilute antibodies. HRP signal was detected using Clarity Western ECL substrate (Bio-Rad). Primary and secondary antibodies and dilution conditions are in Supplemental Table S7.

### Centrosome affinity purification by CAPture

CAPture was conducted as described previously (Carden et al. 2023). Briefly, streptavidin-coated magnetic beads (Dynabeads M-280 Streptavidin; Invitrogen) were washed three times in TBS-N (TBS with 0.1% [v/v] NP-40) and once in CAPture buffer (50 mM Tris-HCl at pH 8.0, 300 mM NaCl, 0.2% [v/v] NP-40, 10% [v/v] glycerol, protease, and phosphatase inhibitors [Thermo Scientific A32961]). Biotinylated peptide (Biotin-SPSPTGGRALRFDPTAFVKAKERKQREIQMKQQ; synthesized >95% purify in Biomatik) was added at 150 ng (30 µL of 5 mg/mL stock solution) per pull-down to M-280 beads in CAPture buffer and placed on rotating wheel at 12 rpm for 1.5 h at 4°C. Expi293F cells (3.6 × 10^8^ per pull-down) were lysed in CAPture buffer for 30 min on ice, sonicated at 700 W in an ultrasonic processor for 10 3 sec pulses at 30% amplitude with a 3 mm microtip probe (Fisher Scientific 418-21), and spun at 2500*g* for 10 min at 4°C. Supernatant was incubated with peptide-bound beads on a rotating wheel at 12 rpm for 1 h at 4°C. After incubation, beads were washed four times with CAPture buffer and twice with 100 mM NH_4_HCO_3_ before being snap-frozen and stored at −80°C.

### On-bead tryptic digestion, phosphopeptide enrichment, and LC-MS/MS analysis

Beads were resuspended in 18 µL of 20 mM Tris-HCl (pH 8.0), 0.8 µL of 100 mM dithiothreitol was added, and disulfide bonds were reduced by incubation for 30 min at room temperature. Free cysteines were alkylated by the addition of 1.2 µL of 100 mM iodoacetamide followed by incubation in the dark for 10 min. Five-hundred nanograms of trypsin (Promega V5113) was added, and proteins were digested overnight at 37°C. Digestion was stopped by addition of formic acid to a final concentration of 2%. Peptides were desalted using C18 ZipTip columns (Millipore ZTC18S096). Five percent of the digested sample was reserved for total protein analysis; the rest was subjected to phosphopeptide enrichment using PTMScan Phospho-Enrichment IMAC Fe-NTA magnetic beads (Cell Signaling Technology 20432S).

Total peptides and phosphopeptide-enriched samples were analyzed using online liquid chromatography coupled with tandem mass spectrometry (LC-MS/MS). The experiment was performed using a NanoAcquity UPLC system (Waters) connected to an Orbitrap Fusion Lumos mass spectrometer (Thermo Scientific). A binary solvent system was used, consisting of 0.1% formic acid in water (solvent A) and 0.1% formic acid in acetonitrile (solvent B). Chromatographic separation was performed using an Easy-Spray HPLC column (75 µm × 150 mm; Thermo Scientific) at a flow rate of 300 nL/min. A linear gradient elution was applied, increasing solvent B from 5% to 30% over 132 min for total protein samples or over 72 min for phosphopeptide-enriched samples. For data-dependent MS/MS acquisition, precursor ions were measured in the Orbitrap over an *m/z* range of 375–1500 at a resolution of 120,000 FWHM (cycle time: 3 sec, maximum injection time: 50 msec, intensity threshold: 2 × 10^4^). Fragment ions generated by higher-energy collisional dissociation (HCD) were detected in the Orbitrap with a resolution of 30,000 FWHM (collision energy: 30%, quadrupole isolation window: 1.6 *m/z*, maximum injection time: 100 msec).

The acquired raw mass spectrometry data were converted to peak lists using in-house PAVA software, followed by analysis using Protein Prospector (version 6.6.5) (Chalkley et al. 2008). Data were searched against a concatenated database of human entries from the SwissProt database (downloaded January 2024) and sequence-randomized decoy entries, employing precursor and fragment mass tolerances of ±10 and ±20 ppm, respectively. Carbamidomethylation of cysteine residues was set as a constant modification, while variable modifications included N-terminal acetylation, N-terminal methionine acetylation and oxidation, pyroglutamate formation from N-terminal glutamine, loss of the protein N-terminal methionine, N-terminal methionine excision followed by acetylation of the new N terminus, oxidation of methionine residues, and phosphorylation of serine, threonine, or tyrosine residues (for the phosphopeptide-enriched samples only). Results were thresholded at a false discovery rate (FDR) of 1% at both the protein and peptide levels based on target:decoy database searching.

For label-free quantification of phosphopeptides and proteins, results were output in .blib format and then imported into Skyline for MS1 filtering (Schilling et al. 2012). Data processing, normalization, and statistical analysis were carried out using the workflow based on qPLEXanalyzer and limma package from Bioconductor (Ritchie et al. 2015; Papachristou et al. 2018).

### Transmission electron microscopy of RPE1 cells

For TEM, cells were plated on 8 well Permanox slides (Nunc 177445), gently washed in PBS (no calcium and no magnesium), and fixed in 3.5% EM-grade glutaraldehyde (Electron Microscopy Sciences 16210) in PBS for 10 min at 37°C. Fixative was removed and replaced with fresh fixative, and samples were incubated for 1 h at 4°C. Slides were washed three times with 0.1 m PB and processed for TEM as described previously (Singla et al. 2010).

### Statistical analysis

All statistical testing was performed using Prism (v.9.5.1). All data used for statistical analysis are in Supplemental Table S8. Statistical tests used for each experiment are listed in the accompanying figure legends. Significant differences were considered as nonsignificant (ns), *P* < 0.05 (*), *P* < 0.01 (**), *P* < 0.001 (***), or *P* < 0.0001 (****) by Student's *t*-test, by *F*-test of equal variance, by one-way analysis of variance followed by post hoc tests (Dunn's multiple comparisons or linear trend analysis), or by two-way ANOVA followed by Šídák's multiple comparison as detailed in the figure legends.

## Supplemental Material

Supplement 1

Supplement 2

Supplement 3
