# Supplementary material for: CDK1 and CEP97 cooperatively control centriole length to orchestrate ciliogenesis and developmental patterning
Source: Genes Dev. 2026 Jul 1;40(13-14):1133–51. doi: 10.1101/gad.353426.125 (PMC13322075; doi:10.1101/gad.353426.125)
Supplement: Supplement 1 [file Supplemental_Figures.docx]

**
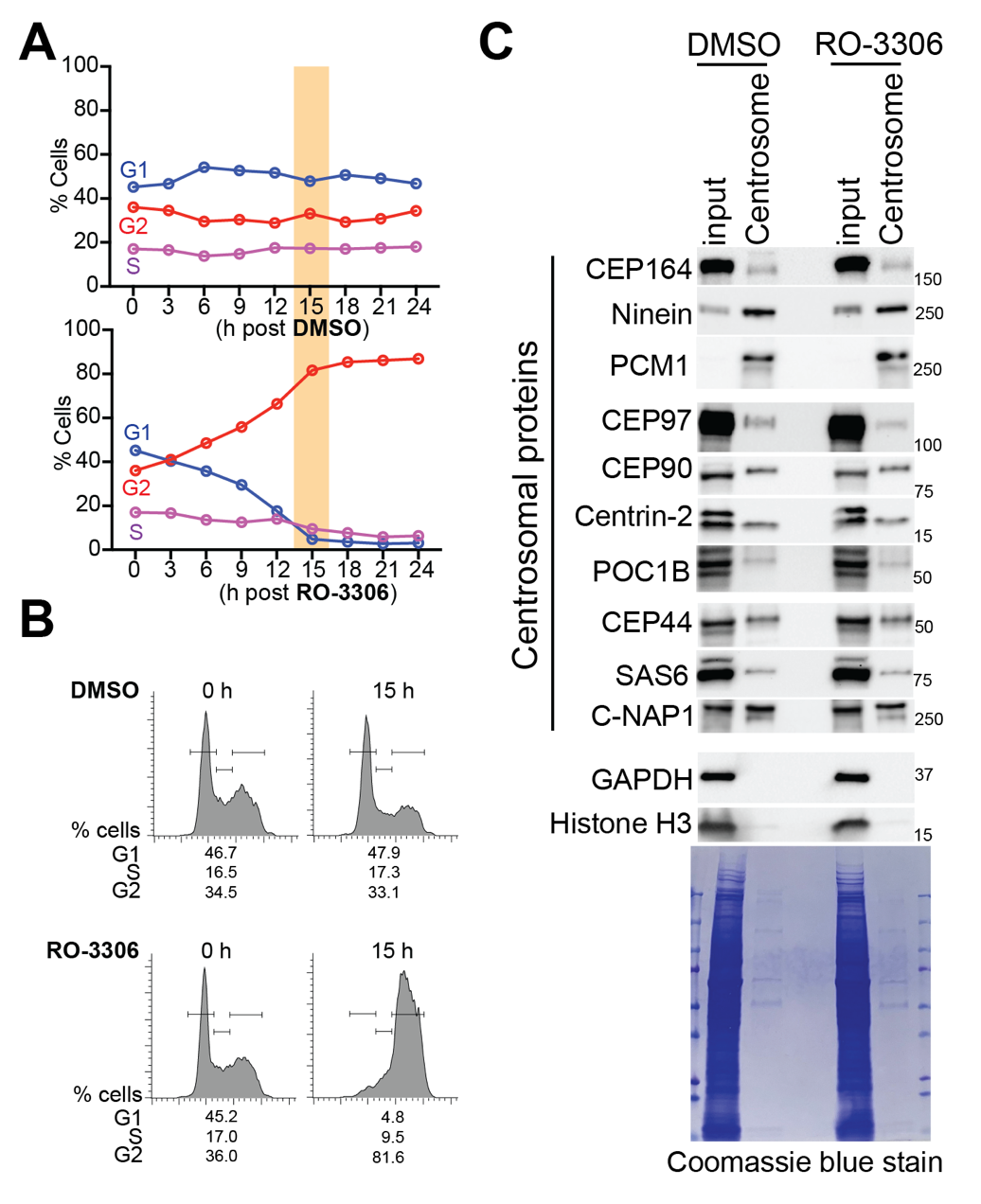
**

**Figure S1. CAPture purified centrosome from DMSO or RO-3306-treated Expi293F cells.**

**(A)** DNA content analysis of Expi293F cells at indicated hours post RO-3306 or DMSO treatment.

**(B)** Representative DNA content analysis of Expi293F cells at 0-, or 15-hour post RO-3306 or DMSO treatment.

**(C)** CAPture purified centrosome from DMSO or RO-3306 treated Expi293F cells. Top, purified centrosome was immunoblotted for indicated proteins. Bottom, Coomassie blue stain of the input or purified centrosome.

**
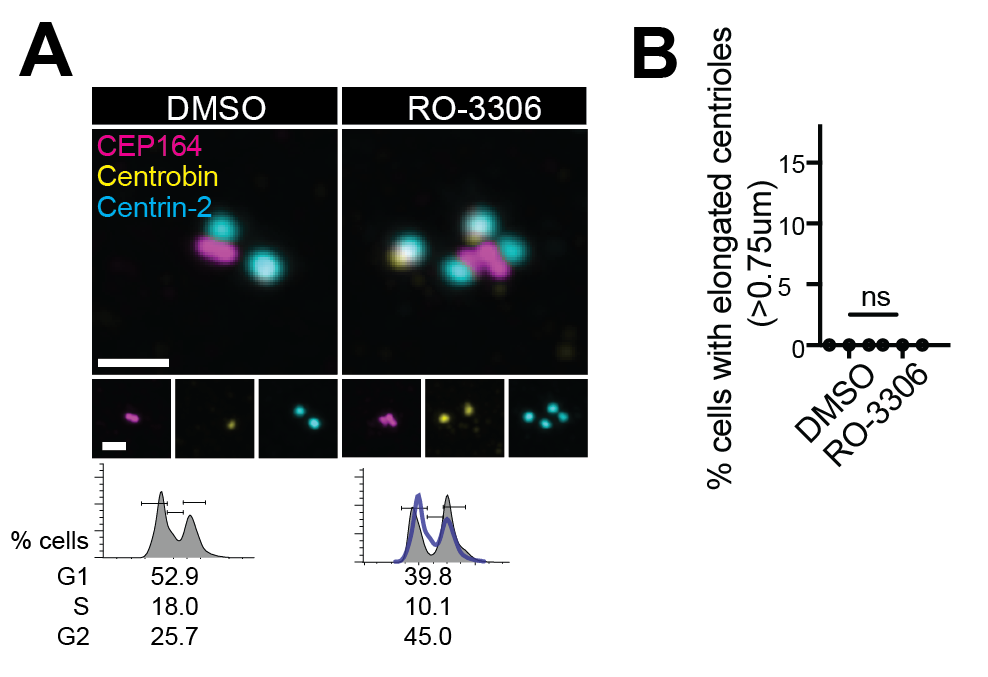
**

**Figure S2. CDK1 inhibition does not alter centriole length in wild-type RPE1 cells.**

**(A)** Immunofluorescence imaging of wild-type RPE1 cells treated with DMSO or RO-3306, and stained for CEP164 (centriole, magenta), Centrobin (centriole, yellow), and Centrin-2 (centriole, cyan). Insets show individual channels. Bottom, DNA content analysis of cells treated with indicated drugs. Scale bars, 1 μm.

**(B)** Quantification of the percentage of cells with elongated centrioles in drug-treated wild-type RPE1 cells as in (A). n=3 biological replicates, with 50-100 cells per replicate. Statistical significance was assessed by student’s t-test in (B). A p value less than 0.05 was considered statistically significant and is denoted as follows: *<0.05, **<0.01, ***<0.001, and ****<0.0001. Data are represented as means ± SD.

**
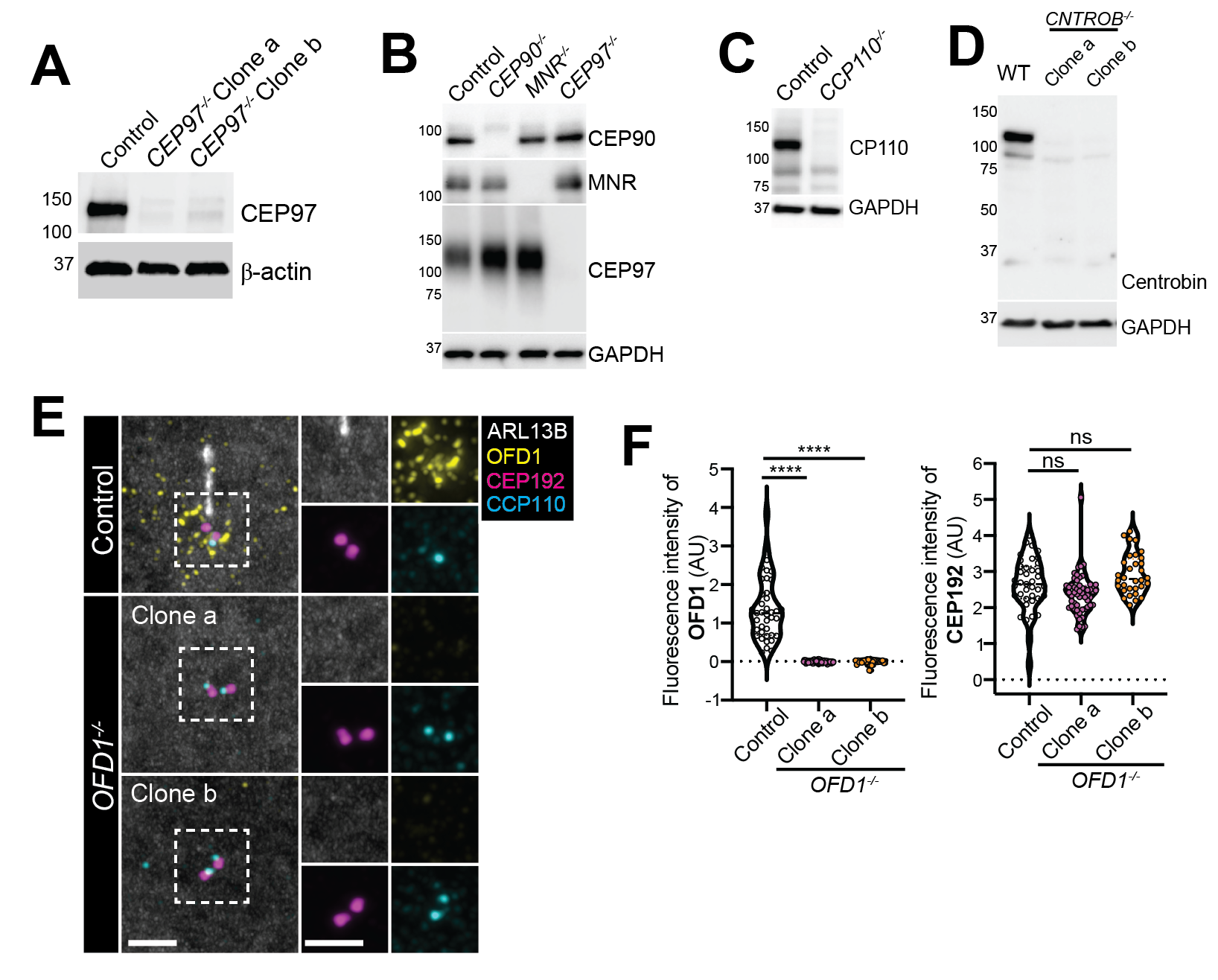
**

**Figure S3. Knockout validations in RPE1 cells.**

**(A-D)** Whole cell lysates derived from wild-type, *CEP97^-/-^*, *CEP90^-/-^*, *MNR^-/-^*, *CCP110^-/-^, CNTROB^-/-^* RPE1 cells were immunoblotted for indicated proteins.

**(E)** Immunofluorescence images of wild-type and *OFD1^-/-^* RPE1 cells were serum-starved for 48 hours, and stained for ARL13B (grey), OFD1 (yellow), CEP192 (magenta), and CCP110 (cyan). Scale bar, 2 μm.

**(F)** Quantification of the fluorescence intensities of OFD1 and CEP192 from wild-type and *OFD1^-/-^* RPE1 cells, as in (E). Insets show magnification of centrioles in boxed regions. n=30-50 cells per cell line. Significance was determined via one-way ANOVA followed by Tukey’s multiple comparison tests (F). A p value less than 0.05 was considered statistically significant and is denoted as follows: *<0.05, **<0.01, ***<0.001, and ****<0.0001. Data are represented as means ± SD.

**
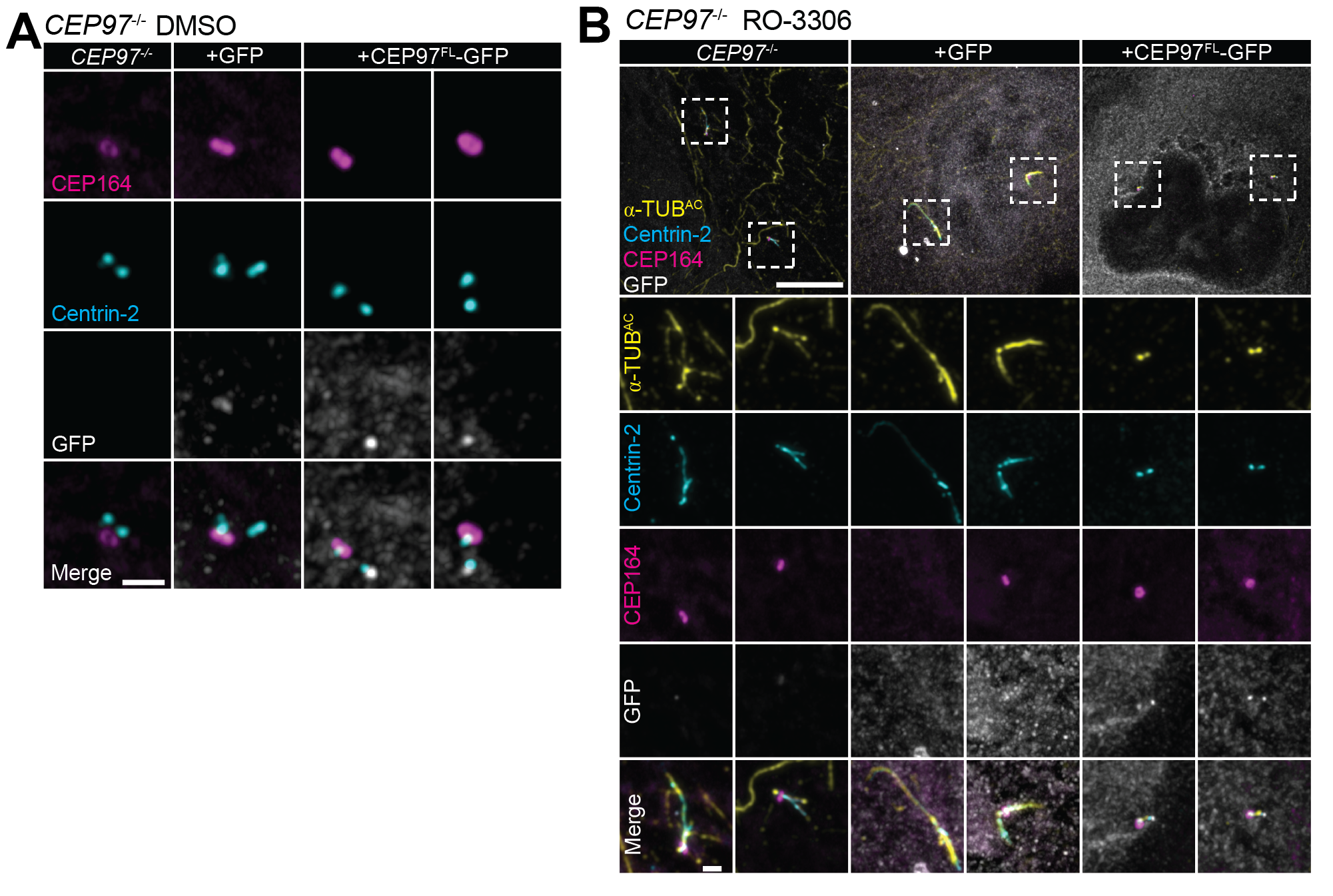
**

**Figure S4. Re-expressed full-length CEP97 localizes to centrioles and restricts centriole length in *CEP97^-/-^* cells.**

**(A)** Immunofluorescence imaging of *CEP97^-/-^* RPE1 cells stably expressing GFP or CEP97-GFP. Cells were serum-starved and stained for CEP164 (centriole, magenta), Centrin-2 (centriole, cyan), and GFP (grey). Scale bars, 1 μm.

**(B)** Parental *CEP97^-/-^* RPE1 cells, or *CEP97^-/-^* RPE1 stably expressing GFP or CEP97-GFP, were treated with RO-3306 for 24 hours and stained for α-Tub^AC^ (centriole, yellow), Centrin-2 (centriole, cyan), CEP164 (centriole, magenta), and GFP (grey). Insets show magnification of centrioles in boxed regions. Scale bars, 10 μm and 1 μm (inset).

**
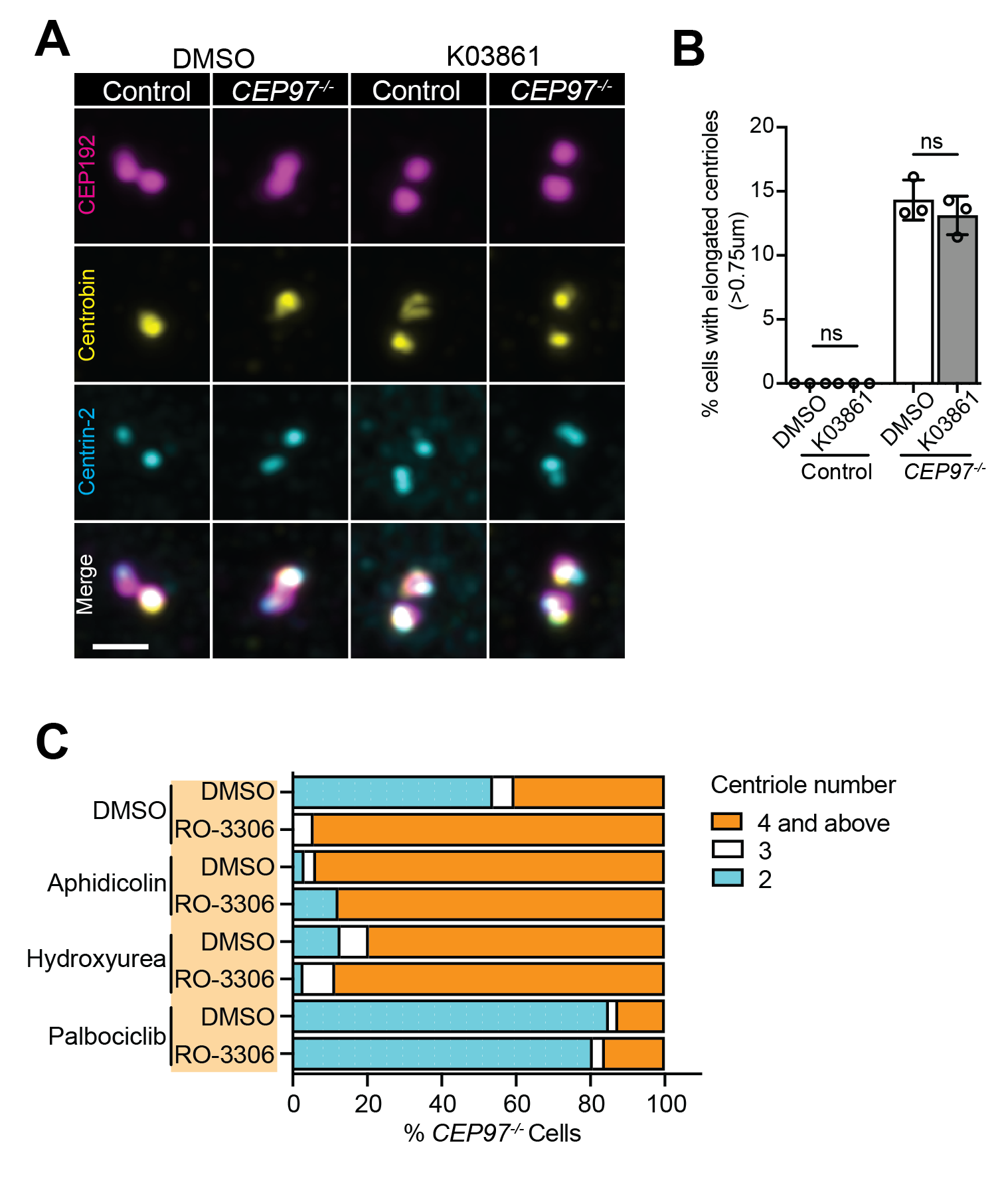
**

**Figure S5. Effect of cell cycle inhibitors on centriole length and number.**

**(A)** Immunofluorescence imaging of wild-type and *CEP97^-/-^* RPE1 cells treated with DMSO or K03861, a selective CDK2 inhibitor, for 24h and stained for CEP192 (centriole, magenta), Centrobin (centriole, yellow), and Centrin-2 (centriole, cyan). Scale bars, 1 μm.

**(B)** Quantification of the percentage of cells with elongated centrioles in drug-treated wild-type and *CEP97^-/-^* RPE1 cells as in (A). n=3 biological replicates, with 50-100 cells per replicate.

**(C)** Quantification of the percentage of *CEP97^-/-^* RPE1 cells with indicated number of centrioles. Cells were treated with indicated drugs, as in Figure 3D. Data combine results from 3 biological replicates, with 200-300 cells per condition. Statistical significance was assessed by two-way ANOVA followed by Šídák's multiple comparison test (B). A p value less than 0.05 was considered statistically significant and is denoted as follows: *<0.05, **<0.01, ***<0.001, and ****<0.0001. Data are represented as means ± SD.

**
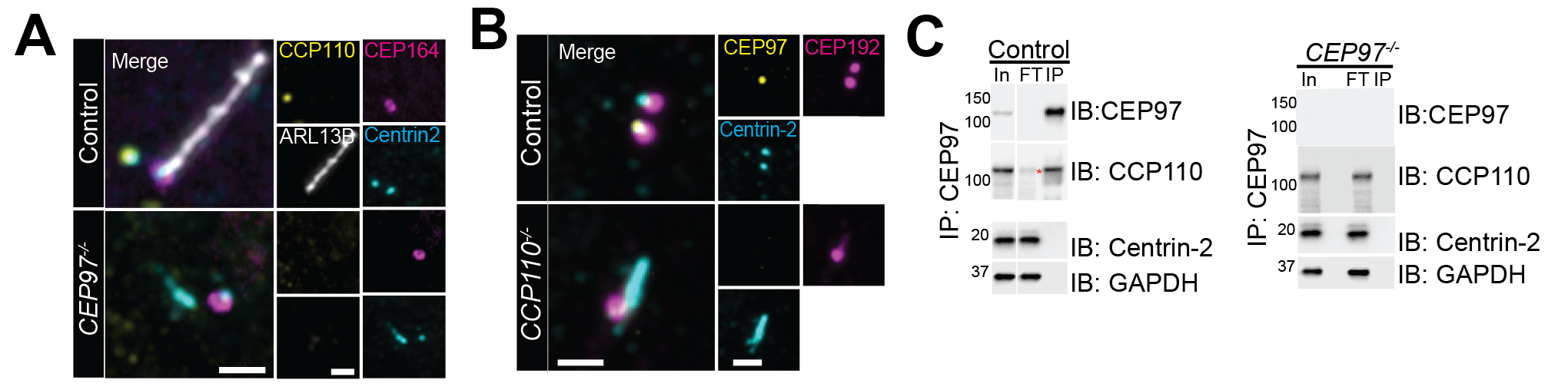
**

**Figure S6. CEP97 and CCP110 are required for the centriolar localization of each other.**

**(A)** Immunofluorescence imaging of serum-starved wild-type and *CEP97^-/-^* RPE1 cells, stained for CEP164 (centriole, magenta), CCP110 (centriole, yellow), and Centrin-2 (cyan). Scale bar, 1 μm.

**(B)** Immunofluorescence imaging of serum-starved wild-type and *CCP110^-/-^* RPE1 cells, stained for CEP192 (magenta), CEP97 (yellow), and Centrin-2 (cyan). Scale bar, 1 μm.

**(C)** Immunoblot of CEP97-CCP110 co-immunoprecipitation. CEP97 was immunoprecipitated (IP) from whole cell lysate derived from wild-type and *CEP97^-/-^* RPE1 and immunoblotted for indicated proteins. In, input. FT, flow through.

**
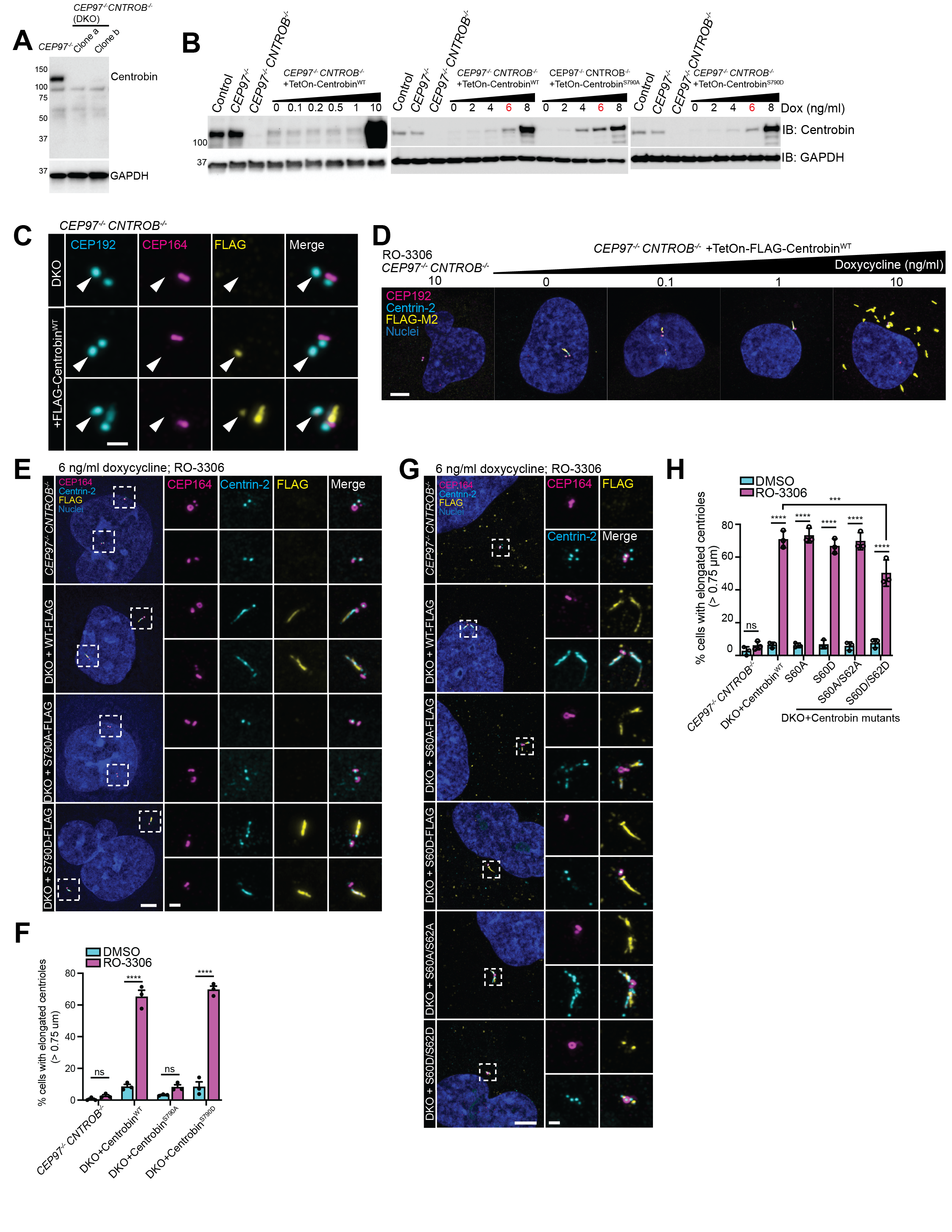
**

**Figure S7. Re-expressing Centrobin restores centriole over-elongation in *CEP97^-/-^ CNTROB^-/-^* cells.**

**(A)** Whole cell lysates derived from wild-type, *CEP97^-/-^CNTROB^-/-^* RPE1 cells were immunoblotted for indicated proteins.

**(B)** RPE1 cells of indicated genotypes were treated with indicated concentrations of doxycycline (Dox). Whole cell lysates were derived and immunoblotted for indicated proteins. Note that levels of Centrobin expression induced by 6 ng/ml of Dox was comparable to endogenous levels of Centrobin.

**(C)** Immunofluorescence imaging of DKO and DKO stably expressing FLAG-Centrobin RPE1 cells, cultured in 6 ng/ml Dox, serum starved for 48 hours, and stained for CEP192 (centriole, cyan), CEP164 (centriole, magenta), and FLAG (yellow). Scale bars, 1 μm.

**(D)** Immunofluorescence imaging of DKO and DKO stably expressing FLAG-Centrobin RPE1 cells cultured in indicated concentration of Dox, treated with RO-3306 for 24 hours, and stained for CEP192 (centriole, magenta), Centrin-2 (centriole, cyan), FLAG (yellow), and Hoechst (nuclei, blue). Scale bars, 5 μm.

**(E)** Immunofluorescence imaging of DKO and DKO stably expressing FLAG-Centrobin RPE1 cells cultured in 6 ng/ml Dox and treated with RO-3306 for 24 hours, and stained for CEP164 (centriole, magenta), Centrin-2 (centriole, cyan), FLAG (yellow), and Hoechst (nuclei, blue). Scale bars, 5 μm and 1 μm (inset).

**(F)** Quantification of the percentage of indicated RPE1 cells with elongated centrioles treated as in (E). n=3 biological replicates, with 50-100 cells per replicate. Statistical significance was assessed by two-way ANOVA followed by Šídák's multiple comparison test (F).

**(G)** Immunofluorescence imaging of DKO RPE1 cells and DKO cells stably expressing FLAG-Centrobin cultured in 6 ng/ml Dox and treated with RO-3306 for 24 hours, and stained for CEP164 (centriole, magenta), Centrin-2 (centriole, cyan), FLAG (yellow), and Hoechst (nuclei, blue). Scale bars, 1 μm.

**(H)** Quantification of the percentage of indicated RPE1 cells with elongated centrioles treated as in (G). n=3 biological replicates, with 50-100 cells per replicate. Statistical significance was assessed by two-way ANOVA followed by Šídák's multiple comparison test (F,H). A p value less than 0.05 was considered statistically significant and is denoted as follows: *<0.05, **<0.01, ***<0.001, and ****<0.0001. Data are represented as means ± SD.

**
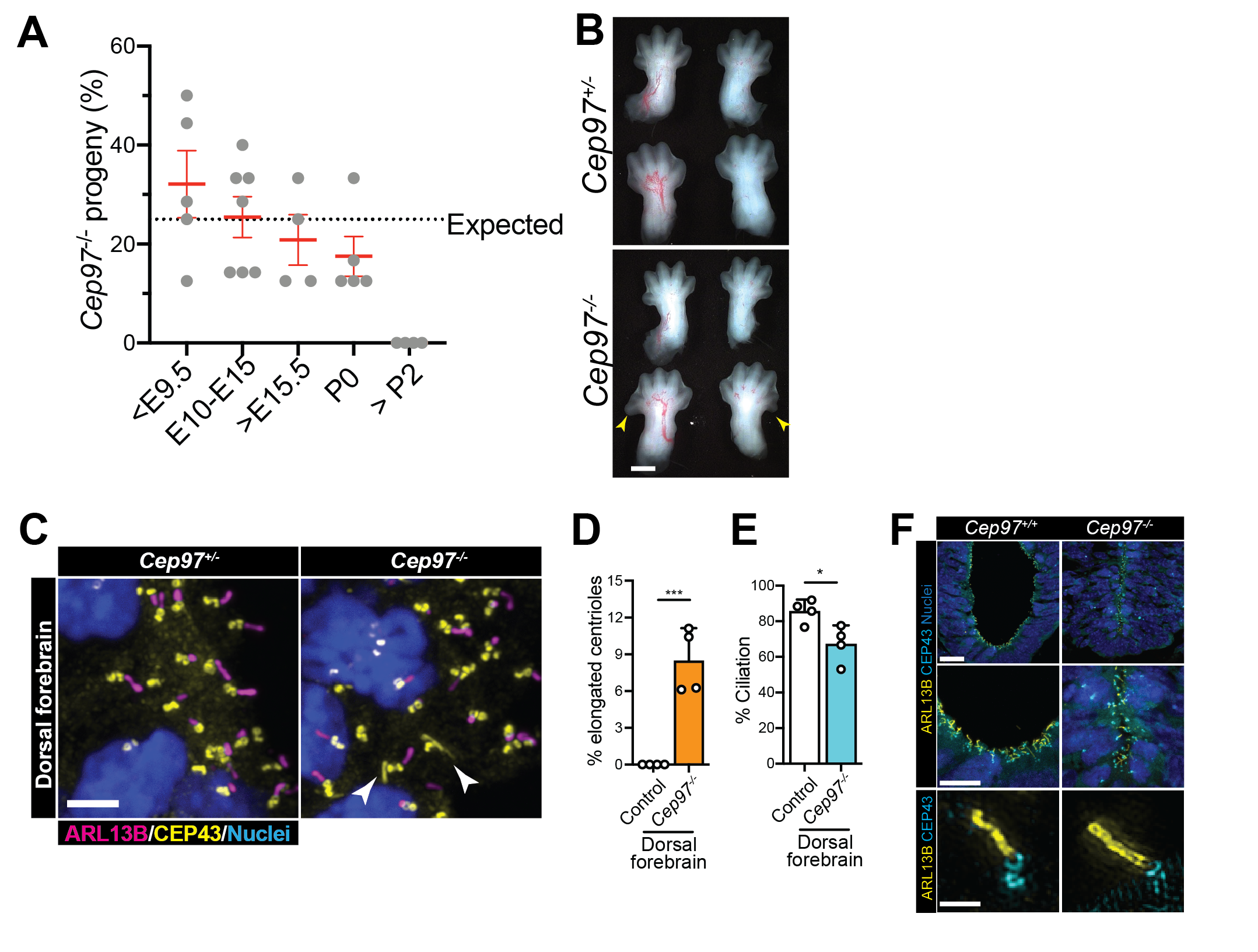
**

**Figure S8. *Cep97^-/-^* mice exhibit neonatal lethality and multiple developmental abnormalities.**

**(A)** Percentage of *Cep97^-/-^* embryos recovered from a heterozygous breeding scheme (*Cep97^+/-^* crossed with *Cep97^+/-^*).

**(B)** Forelimbs and hindlimbs from control and *Cep97^-/-^* embryos at E14.5. Arrowheads indicate preaxial polydactyly. Scale bar, 1mm.

**(C)** Dorsal forebrain of E11.5 *Cep97^-/-^* and littermate, stained for ARL13B (cilia, magenta), CEP43 (centrioles, yellow) and Hoechst (nuclei, blue). Arrowheads indicate examples of elongated centrioles. Scale bars, 3 μm.

**(D,E)** Quantification of the percentage of cells with elongated centrioles (D) and percentage ciliation (E) in the dorsal forebrain of E11.5 control and *Cep97^-/-^* embryos, stained as in (C). n=4 embryos per group.

**(F)** Immunofluorescence imaging of neural tubes derived from *Cep97^-/-^* and littermate control mouse embryos at E9 and stained for ARL13B (cilia, yellow), CEP43 (centriole, cyan), and Hoechst (nuclei, blue). Scale bars, 20 μm (top), 10 μm (middle), and 1 μm (bottom).

**
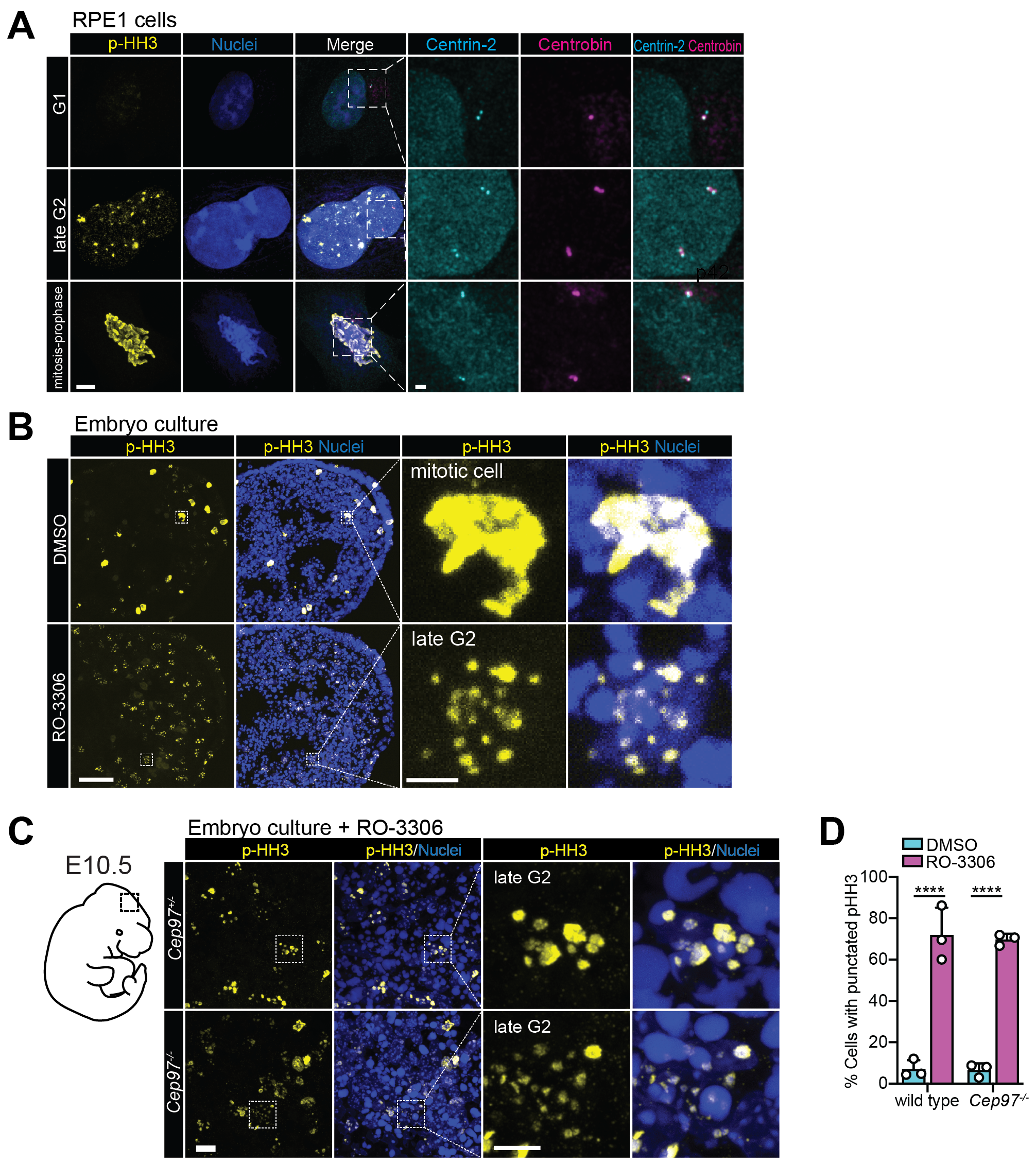
**

**Figure S9. RO-3306 inhibits CDK1 in cells and in mouse embryos.**

**(A)** Immunofluorescence images of wild-type RPE1 cells at different stages of the cell cycle, and stained for p-HH3 (yellow), Centrin-2 (cyan), Centrobin (magenta), and Hoechst (nuclei, blue). Insets show magnification of centrioles in boxed regions. Note cells at the late G2 stage exhibit punctate p-HH3 staining. Scale bars, 5 μm and 1 μm (inset).

**(B)** Immunofluorescence images of the first branchial arch derived from *ex vivo* cultured E10.5 wild-type mouse embryos treated with DMSO or RO-3306 for 24 hours, and stained for p-HH3 (yellow), and Hoechst (nuclei, blue). Insets show magnification of p-HH3 staining in boxed regions. Note cells at the late G2 stage exhibit punctate p-HH3 staining. Scale bars, 50 μm and 5 μm (inset).

**(C)** Left, schematic of E10.5 mouse embryo. Dotted box indicate the brain region where tissue were stained and imaged. Right, immunofluorescence images of the brain derived from *ex vivo* cultured E10.5 *Cep97^-/-^* mouse embryos, treated with RO-3306 for 24 hours, and stained for p-HH3 (yellow), and Hoechst (nuclei, blue). Insets show magnification of p-HH3 in boxed regions. Scale bars, 10 μm and 5 μm (inset).

**(D)** Quantification of the percentage of cells with punctate pHH3 staining in DMSO or RO-3306-treated control and *CEP97^-/-^* embryos (C). n=3 biological replicates, with 50-100 cells per replicate. Statistical significance was assessed by two-way ANOVA followed by Šídák's multiple comparison test (D). A p value less than 0.05 was considered statistically significant and is denoted as follows: *<0.05, **<0.01, ***<0.001, and ****<0.0001. Data are represented as means ± SD.

**
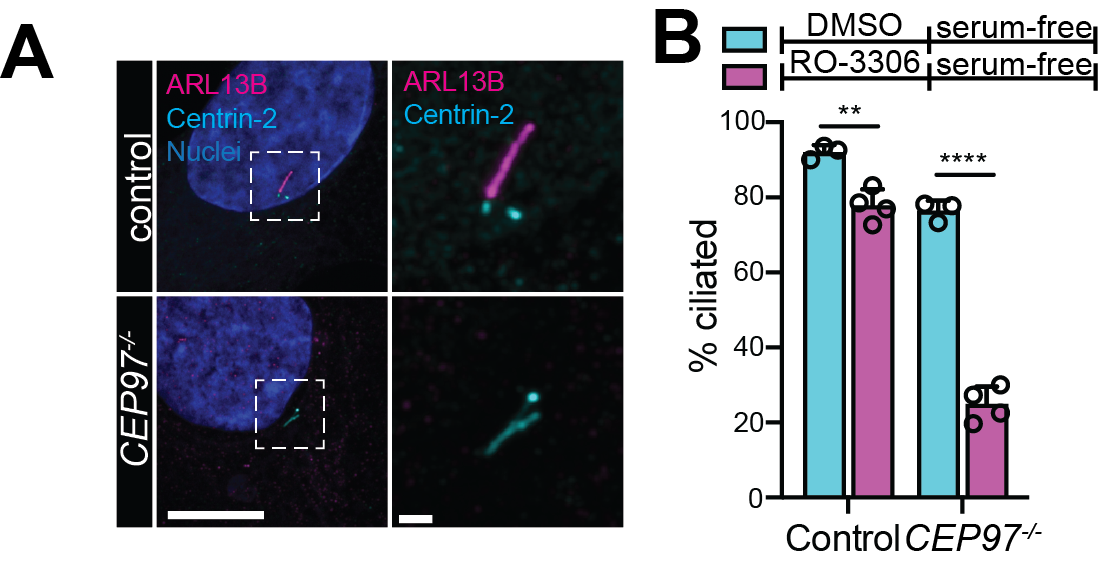
**

**Figure S10. CDK1 inhibition impairs ciliogenesis in *CEP97^-/-^* RPE1 cells.**

**(A)** Immunofluorescence imaging of wild-type and *CEP97^-/-^* RPE1 cells treated with DMSO or RO-3306 for 24 hours, serum starved for 48 h and stained for ARL13B (cilia, magenta), Centrin-2 (centriole, cyan) and Hoechst (nuclei, blue). Scale bars, 5 μm and 1 μm (inset).

**(B)** Quantification of ciliation percentage of indicated RPE1 cells treated as in (A). n=3 biological replicates, with 50-100 cells per replicate. Statistical significance was assessed by two-way ANOVA followed by Šídák's multiple comparison test (B). A p value less than 0.05 was considered statistically significant and is denoted as follows: *<0.05, **<0.01, ***<0.001, and ****<0.0001. Data are represented as means ± SD.

**
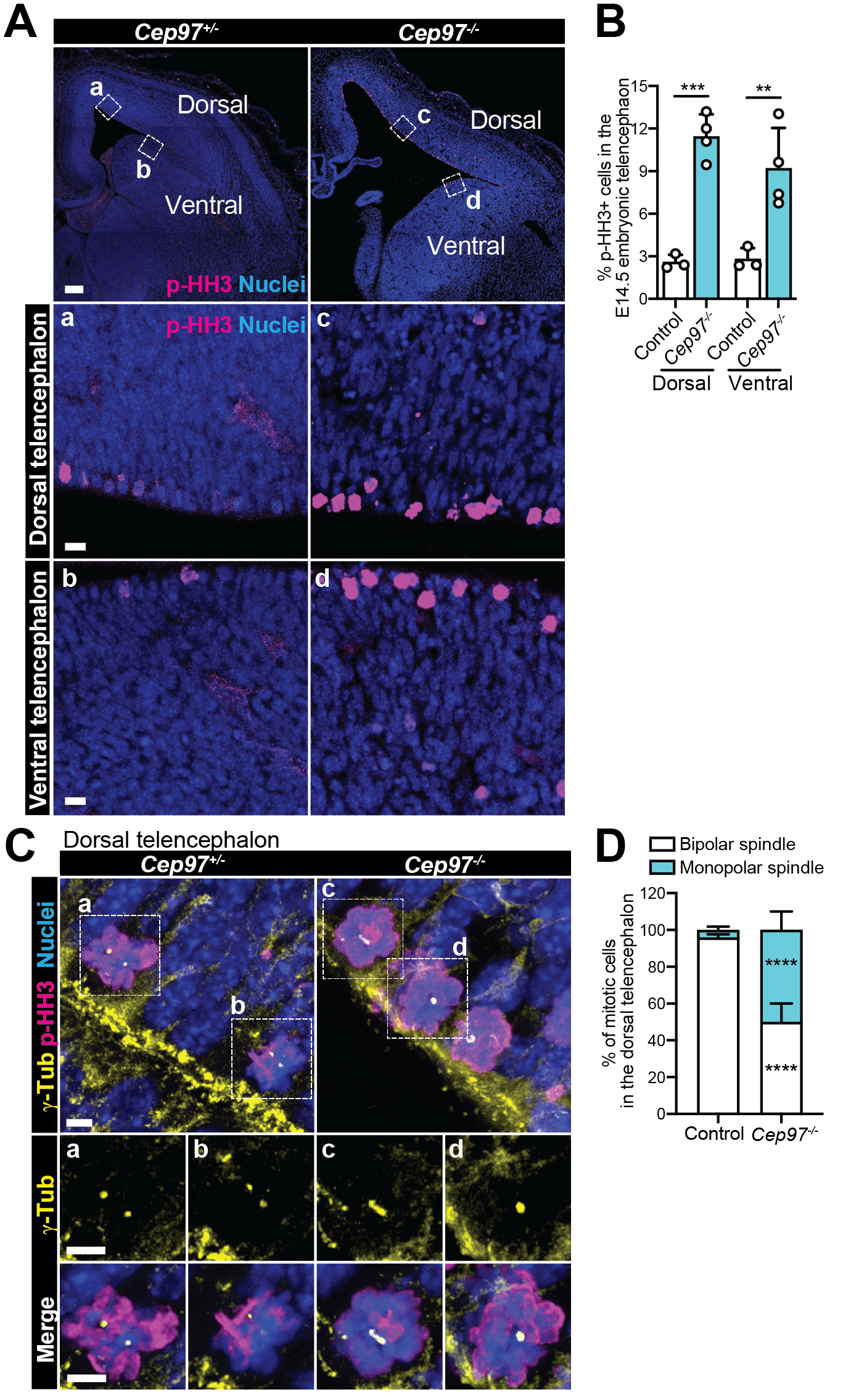
**

**Figure S11. CEP97 contributes to bipolar spindle formation and dorsal telencephalon development.**

**(A)** Upper panels, immunofluorescence imaging of coronal telencephalon sections of E14.5 control and *Cep97^-/-^* embryos, stained for p-HH3 (magenta), Hoechst (nuclei, blue). Lower panels, insets show magnified views of the indicated parts of the dorsal and ventral telencephalons. Scale bars, 100 μm and 10 μm (inset).

**(B)** Quantification of the percentage of p-HH3 positive mitotic cells in the dorsal and ventral telencephalons of E14.5 control and *Cep97^-/-^* embryos, stained as in (A). n=3-4 embryos per group; 600-800 cells per embryo.

**(C)** Upper panel, immunofluorescence imaging of coronal sections of E14.5 control and *Cep97^-/-^* dorsal telencephalons, stained for γ-Tubulin (yellow), p-HH3 (magenta), Hoechst (nuclei, blue). Lower panel, insets show magnified views of the indicated parts of the dorsal telencephalons. Scale bars, 3 μm.

**(D)** Quantification of the percentage of bipolar and monopolar cells at the dorsal telencephalon of E14.5 control and *Cep97^-/-^* embryos, stained as in (C). n=3-4 embryos per group; 40-50 cells per embryo.

**
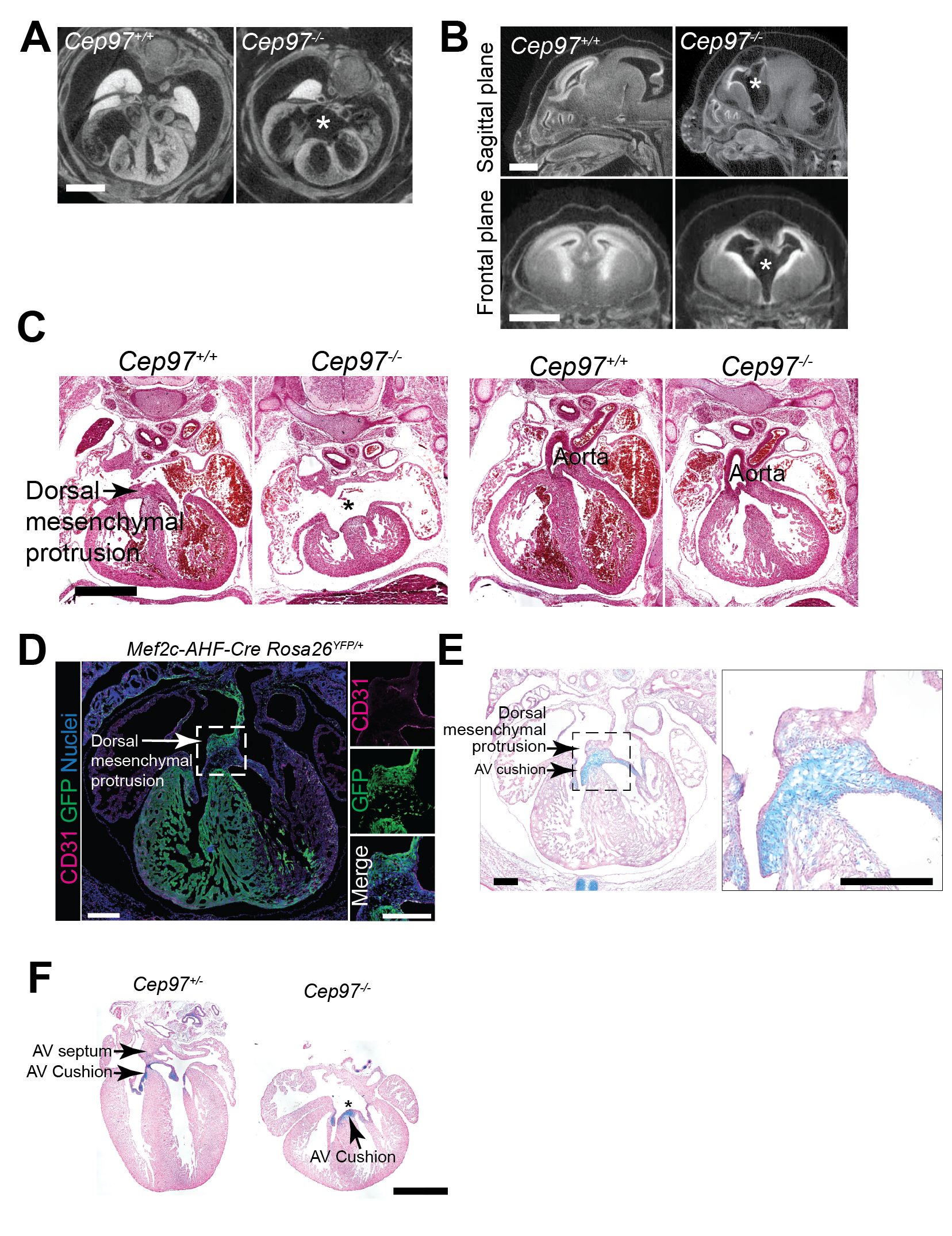
**

**Figure S12. CEP97 is required for heart and brain development.**

**(A-B)** Micro-computed tomography of E17.5 wild-type and *Cep97^-/-^* embryos, presenting transverse four-chambered-view of the heart (A), and sagittal and frontal planes of the head (B). The atrio-ventricular septum is absent, the cortex layer is thinner, and the ventricles are enlarged in the *Cep97^-/-^* embryo (asterisks). Scale bars, 500 μm (A) and 2 mm (B).

**(C)** Histology of *Cep97^-/-^* and littermate control mouse embryo hearts at E15.5. Note the dorsal mesenchymal protrusion is absent in CEP97 mutant heart, as indicated by asterisk; aorta in CEP97 mutant heart overrides both left and right ventricles. Scale bar, 0.5mm.

**(D)** Immunofluorescence imaging of embryonic heart derived from *Mef2C*-AHF-Cre; *Rosa26^YFP/+^* at E15.5. Images depict transverse plane of embryonic heart stained for CD31 (endothelium, magenta), GFP (Mef2C-AHF lineage, green), and Hoechst (nuclei, blue). Insets show magnification of boxed region. Dorsal mesenchymal protrusion is marked GFP positive by *Mef2C*-AHF-Cre. Scale bars, 250 μm.

**(E)** Alcian blue stain of embryonic heart derived from *Mef2C*-AHF-Cre; *Rosa26^YFP/+^* at E15.5, consecutive sections from (D), counter stained with nuclear fast red. Note that alcian blue marks the atrio-ventricular cushion cells but not the dorsal mesenchymal protrusion. Scale bars, 250 μm.

**(F)** Alcian blue stain of *Cep97^-/-^* and littermate control mouse hearts at P0, counter stained with nuclear fast red. Asterisks indicate AVSD. Scale bar, 1mm.

**
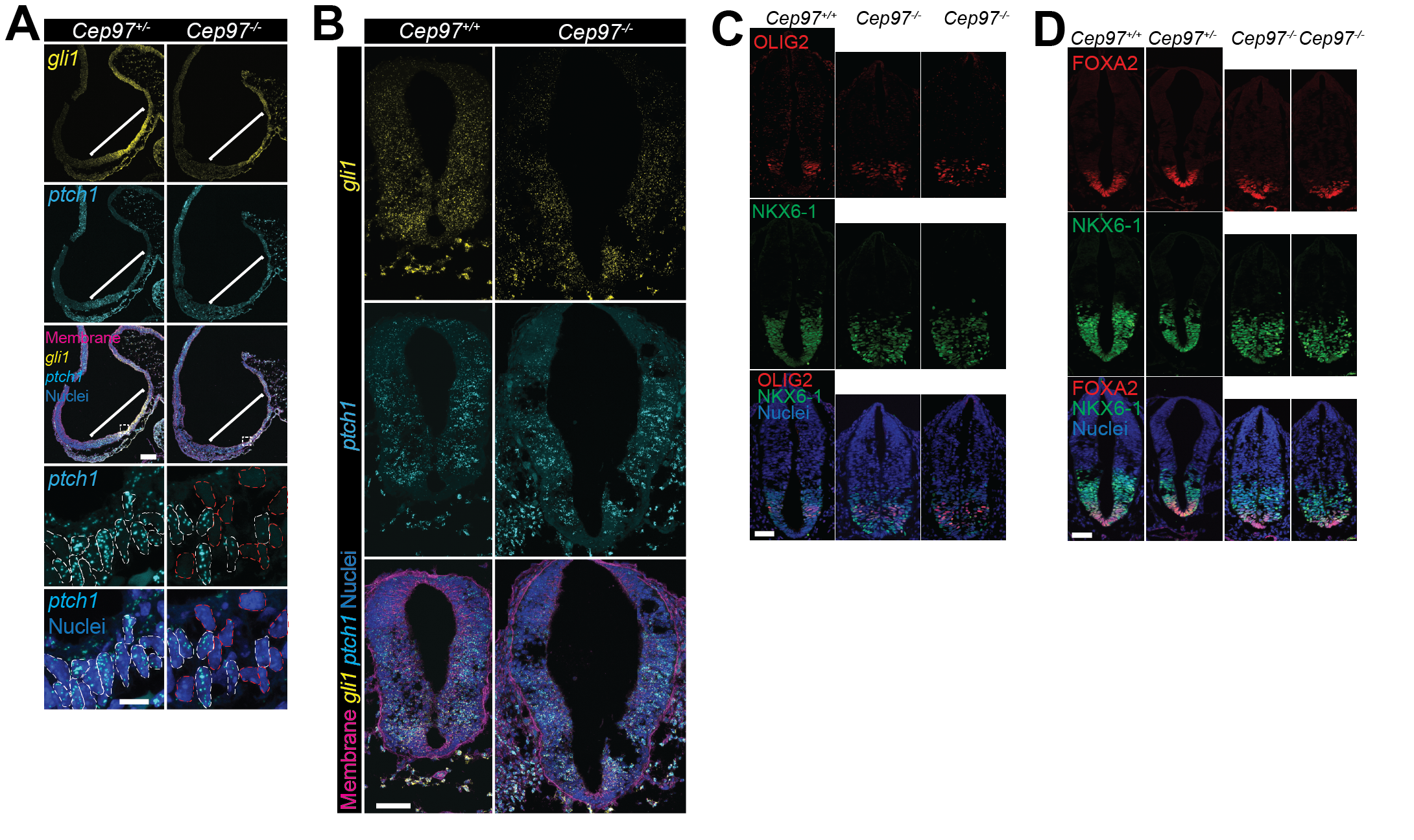
**

**Figure S13. CEP97 is required in select tissues for HH signal transduction and morphogenesis.**

**(A)** Transcripts of HH signaling target genes, *gli1* and *ptch1*, assayed by RNAscope, in the brains of *Cep97^-/-^* and littermate control embryos at E9.5, and stained for Hoechst (nuclei, blue) and Wheat Germ Agglutinin (membrane, magenta). Scale bars, 100 μm and 10 μm (inset).

**(B)** Transcripts of HH signaling target genes, *gli1* and *ptch1*, assayed by RNAscope, in the neural tube of *Cep97^-/-^* and littermate control embryos at E9.5, and stained for Hoechst (nuclei, blue) and Wheat Germ Agglutinin (membrane, magenta). Scale bars, 50 μm.

**(C)** Immunofluorescence imaging of neural tubes derived from *Cep97^-/-^* and littermate control mouse embryos at E9 and stained for OLIG2 (motor neuron progenitor marker, red), Nkx6.1 (pan-ventral marker, green), and Hoechst (nuclei, blue). Scale bars, 50 μm.

**(D)** Immunofluorescence imaging of neural tubes derived from *Cep97^-/-^* and littermate control mouse embryos at E9 and stained for FoxA2 (floor plate, red), Nkx6.1 (pan-ventral marker, green), and Hoechst (nuclei, blue). Scale bars, 50 μm.
